# Estimating immunization coverage at the district level: A case study of measles and diphtheria-pertussis-tetanus-Hib-HepB vaccines in Ethiopia

**DOI:** 10.1371/journal.pgph.0003404

**Published:** 2024-07-25

**Authors:** Latera Tesfaye, Tom Forzy, Fentabil Getnet, Awoke Misganaw, Mesfin Agachew Woldekidan, Asrat Arja Wolde, Samson Warkaye, Solomon Kassahun Gelaw, Solomon Tessema Memirie, Tezera Moshago Berheto, Asnake Worku, Ryoko Sato, Nathaniel Hendrix, Meseret Zelalem Tadesse, Yohannes Lakew Tefera, Mesay Hailu, Stéphane Verguet

**Affiliations:** 1 National Data Management Center for Health, Ethiopian Public Health Institute, Addis Ababa, Ethiopia; 2 Department of Global Health and Population, Harvard T.H. Chan School of Public Health, Boston, Massachusetts, United States of America; 3 Department of Mathematics, Eidgenössische Technische Hochschule (ETH), Zürich, Switzerland; 4 Department of Health Metrics Sciences, University of Washington, Seattle, Washington, United States of America; 5 Policy, Planning, Monitoring, and Evaluation Directorate, Federal Ministry of Health, Addis Ababa, Ethiopia; 6 Addis Center for Ethics and Priority Setting, College of Health Sciences, Addis Ababa University, Addis Ababa, Ethiopia; 7 Maternal, Child & Nutrition Directorate, Federal Ministry of Health, Addis Ababa, Ethiopia; 8 Ethiopian Public Health Institute, Addis Ababa, Ethiopia; PLOS: Public Library of Science, UNITED STATES

## Abstract

Ethiopia has made significant progress in the last two decades in improving the availability and coverage of essential maternal and child health services including childhood immunizations. As Ethiopia keeps momentum towards achieving national immunization goals, methods must be developed to analyze routinely collected health facility data and generate localized coverage estimates. This study leverages the District Health Information Software (DHIS2) platform to estimate immunization coverage for the first dose of measles vaccine (MCV1) and the third dose of diphtheria-pertussis-tetanus-Hib-HepB vaccine (Penta3) across Ethiopian districts (“woredas”). Monthly reported numbers of administered MCV1 and Penta3 immunizations were extracted from public facilities from DHIS2 for 2017/2018-2021/2022 and corrected for quality based on completeness and consistency across time and districts. We then utilized three sources for the target population (infants) to compute administrative coverage estimates: Central Statistical Agency, DHIS2, and WorldPop. The Ethiopian Demographic and Health Surveys were used as benchmarks to which administrative estimates were adjusted at the regional level. Administrative vaccine coverage was estimated for all woredas, and, after adjustments, was bounded within 0–100%. In regions with the highest immunization coverage, MCV1 coverage would range from 83 to 100% and Penta3 coverage from 88 to 100% (Addis Ababa, 2021/2022); MCV1 from 8 to 100% and Penta3 from 4 to 100% (Tigray, 2019/2020). Nationally, the Gini index for MCV1 was 0.37, from 0.13 (Harari) to 0.37 (Somali); for Penta3, it was 0.36, from 0.16 (Harari) to 0.36 (Somali). The use of routine health information systems, such as DHIS2, combined with household surveys permits the generation of local health services coverage estimates. This enables the design of tailored health policies with the capacity to measure progress towards achieving national targets, especially in terms of inequality reductions.

## Introduction

Ethiopia has made significant progress in improving the coverage of essential maternal and child health (MCH) services following the onset of the Millennium Development Goals (MDGs) [1]. The country now aims to further strengthen high-impact and low-cost MCH interventions to meet Sustainable Development Goal 3 (SDG3)–the Health SDG–and its second national Health Sector Transformation Plan (HSTP) targets [2]. Effectively tracking progress toward these objectives requires comprehensively analyzing existing routine health information systems, such as the District Health Information Software 2 (DHIS2) platform [3].

The Ethiopian Expanded Program on Immunization (EPI) targets more than 3 million newborns every year with about 12 antigens [4]. EPI vaccines are routinely administered in around 24,000 health facilities throughout the country. Among these facilities, around 2,700 (11%) did not report delivery of vaccines through the DHIS2 system. Due to initiatives coordinated by the Ethiopian Ministry of Health (MoH) and its partners, immunization levels have considerably increased over time: the percentage of children who received all basic immunizations increased from 17 to 43% between 2000–2019 [5]. According to the 2019 mini Ethiopian Demographic and Health Survey (EDHS), vaccine coverage for the third dose of DPT-hepB-Hib vaccine (the so-called “Penta3”) and the first dose of measles vaccine (MCV1) was 61% and 59%, respectively. However, these levels remain by far insufficient: Ethiopia is one among six countries with the highest number of unvaccinated infants globally [6]. In addition, the disparities in coverage are substantial: from as high as 83% for fully immunized children in the capital city of Addis Ababa to as low as 20% for the mostly pastoralist Afar region; from around 57% in urban areas to about 37% in rural areas (2019 estimates) [5]. COVID-19 and the ongoing armed conflicts in different parts of the country have severely strained the health system and immunization efforts. Out of 30 countries in the WHO Africa region, 17 (57%) showed interruptions in routine MCH operations during the early times of the pandemic [7], which in many African countries is anticipated to have a large long-term impact as time-sensitive MCH services were not given priority during the pandemic [8]. This is especially true in Ethiopia, where additionally the repercussions of armed conflicts have led to significant damages in healthcare-related infrastructures and disruptions in health services access, including immunizations, even though precise reporting remains difficult [9].

The most widely used source for tracking health services coverage is household surveys, like the DHS, but their frequency (every five years or so) and sample size hamper the systematic generation of annual local estimations [10]. Yet annual estimations of health services coverage at national, subnational, and district (“woreda”) levels are required to evaluate national plans and set priorities [11, 12]. Ethiopia’s HSTP and global initiatives such as the SDGs emphasize the enhancement of digital health technologies [2] and of monitoring health services coverage using routine health information systems [13–15]. Those health information systems, in addition to raising opportunities for novel research drawing from numerous repeated large observations, enable the measurement of trends and patterns to assess the impact of different epidemiological, political, socioeconomic, or environmental events [16]. In addition, data from routine health systems can be used for evaluating programs. At the policy level, the data can be leveraged to ultimately improve resources allocation, services delivery, and thus population health, as has been shown in several African countries [17]. One of the widely used routine health information systems is the District Health Information Software (version 2, or DHIS2), which is an open-source, web-based software platform for data collection, management, and analysis [18]. DHIS2 is the world’s largest Health Information Management System platform, currently used by Ministries of Health in 73 low- and middle-income countries. In Ethiopia, DHIS2 implementation started locally in some parts of the country in 2016, and national data reporting started in 2019 [19].

Despite its great potential, DHIS2 remains largely underutilized for decision-making in sub-Saharan African countries, mostly due to data quality concerns, including, for example, completeness, timeliness, and internal and external consistency [3, 17, 20]. A study conducted in Ethiopia’s Tigray region on the use of health facility data for decision-making indicated that the possible reasons for missed or erroneous reporting were staff shortage, lack of knowledge, or understanding of the reporting tool [21]; it also showed that the major reasons behind data inconsistency were data entry errors, arithmetic errors and lack of emphasis on data accuracy. DHIS2 uses paper-based recording where daily services provided are recorded on specific registers. These registers are then aggregated into monthly reports, which can be highly burdensome for health workers [22].

Coverage estimations of health indicators, including childhood immunizations, need accurate population census numbers (i.e., accurate “denominators”). However, the lack of precise data for denominators, such as pregnancies and live births, especially at the district level, complicates coverage estimations [10]. The availability of these data is particularly limited in Ethiopia, where the commonly used census data are obtained from the Central Statistical Agency (CSA), with its most recent assessment dating from 2007. The population numbers, which are being widely used today are forecasted from this census data and the methods used did not consider the ever-changing underlying demographic characteristics. When such denominators are used along with DHIS2 reports to estimate service coverage, they usually yield overestimates (>100% coverage). If the denominators could be adjusted with other data sources, then DHIS2 could be leveraged to produce high-quality coverage estimates for basic health indicators, at the national, subnational, and woreda levels. This is what we intend to demonstrate here with the case study of immunization in Ethiopia.

In this paper, we develop analytical methods and a systematic approach to estimate MCV1 and Penta3 immunization coverage at the district (woreda) level in Ethiopia using DHIS2, EDHS, and multiple sources of population census data.

## Methods

We estimated MCV1 and Penta3 coverage across all Ethiopian woredas (districts). After estimating administrative coverage, we used EDHS household surveys to adjust for potential overestimations and underestimations for each woreda, which we detail here. This section proceeds in three parts: first, we describe the data sources used; second, we detail our estimation approach; and third, we present the summary findings that we display in the Results section.

### Data sources

DHIS2 has 988 woredas, including special towns and special zones. For the year 2022 these woredas captured a total of 29,509 public and private health facilities among which were 7,380 clinics, 3,777 health centers, 17,903 health posts, and 449 hospitals. We used reports collected from public health facilities (health posts, health centers, and hospitals). Vaccines administered in private health facilities, mostly clinics, represented about 1% of all vaccines administered nationally [23]. In 2021, MCV1 was provided either in clinics (1%), hospitals (3%), health centers (27%), or health posts (69%). Here, we assumed that the number of vaccines administered in the health facilities of a specific woreda pertained to that woreda only.

MCV1 and Penta3 coverage were defined as the percentage of surviving infants who received the first dose of a measles-containing vaccine and the third dose of a pentavalent-containing vaccine (S1 Text). The first data source for our denominator (target population) was the 2020 WorldPop population data with a spatial resolution of 100m by 100m [24]. WorldPop is a collaborative project that uses a combination of machine learning methods, expert opinion and census data to map population settlements and further derive population distribution based on satellite images. By applying zonal statistics we overlaid the 2022 standard administrative boundary data from the Regional Bureau of Finance and Economic Development (BoFED) [25] with the WorldPop data to extract population numbers for each woreda (thereafter named WorldPop data). We extracted WorldPop data for 1,081 woredas. The second denominator data used were the DHIS2 numbers of surviving infants. These numbers were estimated by the MoH from 2014/2015 census data [26] by considering multiple social and demographic factors. The third denominator data were from CSA for 2020 and 2021 [26]. CSA data are the official government source of population data, disaggregated by sex and location (region, zone, or woreda). However, the data did not contain age-disaggregated information and we used a conversion factor to obtain the number of surviving infants (S2 Text, p.1). CSA data had 750 administrative woredas with boundaries defined in 2014. To match the population within CSA boundary to that of BoFED, we interpolated the CSA population with the BoFED boundaries using areal interpolation (S2 Text). In case the population values were underestimated, further adjustments were made depending on population values of DHIS2 and WorldPop.

All monthly reports of MCV1 and Penta3 vaccine doses administered for surviving infants for the years 2017/2018-2021/2022 were extracted from DHIS2 and aggregated at woreda level from all vaccine-delivering health facilities. Using the DHIS2 data and the three population estimates (WorldPop, CSA, or DHIS2; see immediately above), we computed immunization coverage at woreda level. We used EDHS data as a benchmark to adjust for overestimation with administrative MCV1 and Penta3 coverage estimations. The most recent EDHS was conducted in 2019. However, it was a mini-survey with small sample size and few indicators included. To circumvent this challenge, we integrated the 2016 and 2019 EDHS surveys. After integration, we estimated MCV1 and Penta3 coverage for all regions and 432 woredas included in the EDHS by applying the same methods implemented by DHS to estimate immunization coverage [27] (details provided in S2 Text).

### Estimation approach

We proceeded in three steps. First, we conducted a data quality adjustment for DHIS2 data (the so-called “headcount” or “numerator” data). Second, we pursued adjustments to the population sizes from the census data (the “denominator” data) while merging numerators and denominators at the woreda level. Third, we estimated coverage levels and proceeded to further adjustments.

#### Step 1—DHIS2 adjustment

First, we checked the quality of DHIS2 monthly data. Completeness, outliers, and internal consistencies are essential data quality dimensions, which we assessed. We treated the missing values in the monthly woreda-level reports while implementing multiple imputations (S3 Text). Distinguishing between missing values due to non-reporting facilities (despite providing the service) and missing values due to service unavailability was difficult. Regardless, the multiple imputation method was executed for all missing values, assuming missing at random. However, if reports were missing across all woredas for a particular month, then the month would be removed. Similarly, if reports were missing for all months for a particular woreda, then the woreda would be removed. Second, we detected and treated outliers. The WHO defines a single monthly report of a given data element as a “moderate” outlier if it is between 2 and 3 standard deviations (SD) from the mean value for the year, and as an “extreme” outlier if it is more than 3 SDs from the mean value for the year [20]. In this study, we only treated extreme outliers. For detecting outliers, we assumed our values were distributed according to a binomial distribution. Outliers were categorized either below or above the mean. For the outliers above the mean, we first assessed whether the outliers could be linked to reporting accumulation, which occurs when reports for several months are not reported on time, but are accumulated and finally reported together a few months later. This eventually leads to low values for some months before an outlying high value. To detect this situation, we compared the five months after (the outlier) to the five months before using a t-test (with a 5% significance level). When values for the five months before were significantly below the ones for the five months after, we took the difference between outlier and median (the "extra-reporting") and distributed it equally across the five months before. Alternatively, when the t-test failed, we assumed that the high numbers were due to errors in reporting. In that case, we replaced them with the median. In order to detect outliers below the mean, we assumed that our data was following a Poisson distribution. We then selected an arbitrary quantile of the approximated Poisson distribution and used it as a threshold. Since we used 3 standard deviations (SD) above the mean as the threshold on the right side (which corresponds to approximately the 99.9th percentile of a normal distribution), we aimed to have an equivalently unlikely outlier threshold on the low side. Specifically, we chose the 0.1th percentile of our assumed Poisson distribution. To determine this threshold value, we first calculated the mean for each woreda in our data. We then computed the probability that an observation would be less than or equal to the 0.1th percentile of the Poisson distribution with that woreda mean, and classified any observations with a probability below this cut-off as outliers. Third, we checked for consistency over time. WHO recommends that reported values for the reference year be within ±33% of the mean value for the previous three years [20]. We checked over time consistency for monthly reported values of each woreda. When evaluating internal consistency between indicators, WHO recommends that pairs of data elements with presumed equal values be within ±10% of one another [20]. Internal consistency can be assessed in different ways. For instance, the number of reported births at health facilities must be equal to the sum of stillbirths and live births; for vaccine dropout, the third dose of the pentavalent (Penta3) vaccine must be lower than the first dose (Penta1); MCV1 is also expected to be lower than the first dose of pentavalent (“Penta1”) [4, 28, 29]. In our study, we used Penta1 to Penta3 and Penta1 to MCV1 ratio of 1.0–1.5 to check internal consistency for both Penta3 and MCV1 [4, 5, 30]. Additionally, to evaluate the internal consistency of Penta3, we also used the third dose of oral poliovirus vaccine (OPV3) as benchmark: both OPV3 and Penta3 are given at 14 weeks after birth (at the same date) and the values from both indicators are expected to be equal. As such, we assessed if the absolute percentage difference between the two was less than 10%. Reporting accuracy of MCV1 was checked against Penta1 with a range of Penta1 to MCV1 ratio from 1.0–1.5, as a dropout rate over the 9 months after Penta1 is administered is expected [28, 29].

#### Step 2—Denominator adjustment and numerator matching

We merged DHIS2 numerators with our denominators (matching at the woreda level). Woreda administrative boundary data for DHIS2 numerators were not accessible. Hence, we implemented a probabilistic record linkage using Levenshtein distance algorithm for matching woreda names between the numerator and denominator data since districts names were not standardized. To estimate coverage, we needed estimated target populations (“denominators”) for the same list of woredas as for the numerators. When DHIS2 denominators were used, evidently names perfectly matched with the numerators. However, for WorldPop and CSA denominators, there were significant variations in woreda spelling compared with DHIS2 numerators. For instance, the same geographical divisions had different names across datasets or were merged or further divided in one dataset but not in another. The matching was performed for 750 woredas within CSA data and 1,081 woredas within Wordpop data against 988 woredas within DHIS2 data (S4 Text).

#### Step 3—Administrative coverage estimation

First, the adjusted numerators were divided by the denominators to estimate unadjusted administrative coverage for each woreda. We generated three distinct coverage estimations using the three different denominators. A crucial step was to adjust administrative coverage with EDHS coverage. We assumed EDHS MCV1 and Penta3 coverage were of good quality at the regional level and corrections were made with respect to regional coverage estimates. We observed substantially inflated coverage (>100%) for most woredas. In a setup where the quality of the denominators is highly uncertain, woredas with coverage estimates of less than 100% could still have the same error as those with inflated coverages. To address this, the use of EDHS became imperative, as it had the advantage not to depend on a denominator estimate that might be inconsistent with the numerator. We applied the correction for all woredas. A simple approach we could have used is to assume that the ratio between estimated and actual coverage was constant across all woredas within a region. However, this would consider all woredas had proportionally equally poor estimates. Rather, a more robust approach would allow the ratios between estimated and actual values not to be necessarily constant. Our general assumption was that numerators were reliable as opposed to denominators. We observed the distribution in woreda administrative coverage to be bell-shaped and right-skewed, and thus assumed it was gamma distributed. We fitted these distributions by maximum-likelihood estimation [31]. Using EDHS region-level coverage estimates as a gold standard, we created a hypothetical continuous distribution composed of unknown true coverage values for each woreda. One key property of this distribution is that it needed to be bounded within 0–100%. Consequently, a beta distribution was used to model this distribution, with the expectation set at the EDHS region point estimate. The second characteristic of this hypothetical distribution is the order of the woredas within the distribution around the mean value. For this, we assumed that the order of woredas observed in the administrative coverage, modeled by a gamma distribution, would be the same for the beta distribution. Therefore, our approach involved matching our observed distribution of administrative coverage to that simulated beta distribution per percentile (see details in S7 Text).

### Synthesizing the findings

We summarized our three corrected coverage estimations for each denominator (DHIS2, CSA, WorldPop). We computed the absolute differences between paired estimates and defined a threshold of a 20-percentage point difference to label one estimation as reliable (or not). If all three percentage differences were under 20%, we took the median of the three estimations. However, if all three percentage differences were above 20%, the estimation for the woreda would be labelled as unreliable. If the percentage difference was less than 20% only for two denominators, the final value for the woreda would be the mean of the corrected estimation of those two denominators. In short, we tested the consistency among the three denominator data sources (instead of selecting one denominator data source only). The rationale for selecting a 20% threshold was that it corresponded to when district-level coverage estimates and their coverage rankings within a region would remain consistent regardless of the denominator used. Indeed, differences above 20% would lead otherwise to large variations in coverage estimates within a region. This approach helped to account for the denominator-specific variations and their impact on coverage estimation. S5 Text provides more details.

We assessed disparities in the number of vaccines administered relative to the population in need, hence defined by the balance between the number of vaccines administered and the actual target population. We generated a modified Lorenz curve to assess the extent of disparities in the distributions of Penta3 and MCV1 coverage and computed associated Gini indices (S8 Text).

All computations were conducted using R software (version 4.0.2), Python (version 3.8), and Microsoft Excel (2016 version). All data used were anonymous secondary data, most of which are publicly available. We received permission from the Ethiopian Ministry of Health to use DHIS2 data.

### Ethics statement

All data used were anonymous secondary data thus ethical approval was not applicable.

## Results

The 2020 and 2021 CSA data consisted of 750 woredas each, with a total population of around 100,000,000 for each year. After applying the conversion factors (S2 Text), the estimated total number of surviving infants was 3,170,300 (2020) and 3,258,900 (2021). After areal interpolation and merging WorldPop and BoFED boundary data, we obtained a total population of 114,785,900 and 3,883,400 surviving infants for 1,081 woredas. The DHIS2 denominator data included a total of 3,278,700 surviving infants from 988 woredas. The 22,129 public health facilities were grouped per woreda, except for 53 hospitals (representing less than 0.5% of total MCV1/Penta 3 vaccines administered). Table 1 summarizes the number of vaccines administered at national and regional levels.

**Table 1:** Summary indicators for the vaccines administered at the regional level in Ethiopia.

Region	Mean Penta3	Standard deviation Penta3	Mean MCV1	Standard deviation MCV1	Population
Addis Ababa	10,529	912	9,973	1,407	3,949,961
Afar	3,291	265	3,016	334	2,046,136
Amhara	48,695	7,302	45,571	7,834	24,709,115
Benishangul Gumuz	2,469	326	2,299	406	1,264,167
Dire Dawa	1,020	102	935	100	521,609
Gambella	972	134	835	169	633,264
Harari	693	92	648	107	284,658
Oromia	117,417	9,595	109,109	11,707	43,935,122
Sidama	11,721	783	11,298	869	5,121,259
SNNPR	34,532	2,679	33,046	3,033	15,108,732
Somali	13,263	3,323	11,732	2,991	6,941,218
South West	7,096	475	6,712	656	3,702,713
Tigray	13,373	865	12,612	935	6,567,898

Notes: Penta3 denotes the third dose of the diphtheria-pertussis-tetanus vaccine; MCV1 denotes the first dose of the measles vaccine. This table shows the annual mean and standard deviation of the vaccines administered, and the total populations across Ethiopian regions. SNNPR: Southern Nations, Nationalities and Peoples Region.

The integration of 2016 and 2019 EDHS resulted in 3,032 eligible children for all immunizations. Fig A6.1 and A6.2 (S6 Text, pp.1–2) show MCV1 and Penta3 coverage for woredas included in the integrated EDHS dataset.

The completeness of annual facility reporting for MCV1 and Penta3 was 67% (n = 14,826 facilities) and 70% (15,490 facilities). The extent of missing values varied across regions. For Addis Ababa, Dire Dawa, Harari, and Sidama, no missing values were detected. This might indicate strong use of DHIS2 by health facilities in these areas. In other regions: for example, for Afar woredas, missing values for Penta3 ranged from 2 to 9%, and from 2 to 11% for MCV1. For Amhara woredas, missing values for Penta3 ranged from 2 to 25%, and from 2 to 34% for MCV1. Somali had the greatest number of missing values of all regions with multiple missing reports across months. For Penta3, Somali had woredas with a missing value ranging from 2 to 52%, and from 2 to 58% for MCV1. Only 14 out of 98 woredas had no missing values in Somali. Table A6.1 and Fig A6.5–12 (S6 Text, pp-5–10) summarize the extent of missing values per region.

After imputations, the variations between imputed and original values were checked using density plots (see S6 Text, Fig A6.14 for selected woredas). We observed significant outliers after managing the missing values. The extent of outliers varied greatly across regions. Across Tigray woredas, the outlier percentage ranged from 7 to 12% of all monthly reports; and, similarly, for the other regions: 2–5% (Addis Ababa), 3–6% (Afar), 5–12% (Amhara), 3–12% (Benishangul-Gumuz), 5–7% (Dire Dawa), 2–6% (Gambella), 12–24% (Harari), 2–5% (Oromia), 1–8% (Sidama), 6–23% (SNNP), 2–8% (Somali), and 1–9% (SWR) (S6 Text). From 456 woredas with at least one extreme outlier, report accumulation (that is reports from several months are cumulated and the resulting aggregate is reported toward one particular month only) was detected for 120 woredas and the ‘extra-reporting’—the number of vaccines exceeding the annual mean—was distributed across five months before that outlier value. However, for the remaining 336 woredas, median values were replaced. These two management steps led to significant decreases in data variability. Fig A6.15 (S6 Text) shows these distributions before/after data management for selected woredas. In addition, for all woredas, both MCV1 and Penta3 data successfully met the criteria for internal consistency tests.

For each woreda, we obtained a name from DHIS2 with unique matching to the woreda list of WorldPop data (except for three woredas). The match was exact (letter to letter) in 40% of cases, and we manually double-checked the matching with the Levenshtein distance algorithm. After a few corrections, we rapidly yielded 90% correspondence. For the remaining woredas, we proceeded to matching by hand. Additionally, to match DHIS2 woredas with CSA, as the boundary data for CSA were available, we first matched with WorldPop. We used the DHIS2 to WorldPop matching to match CSA with DHIS2.

**Fig 1:**
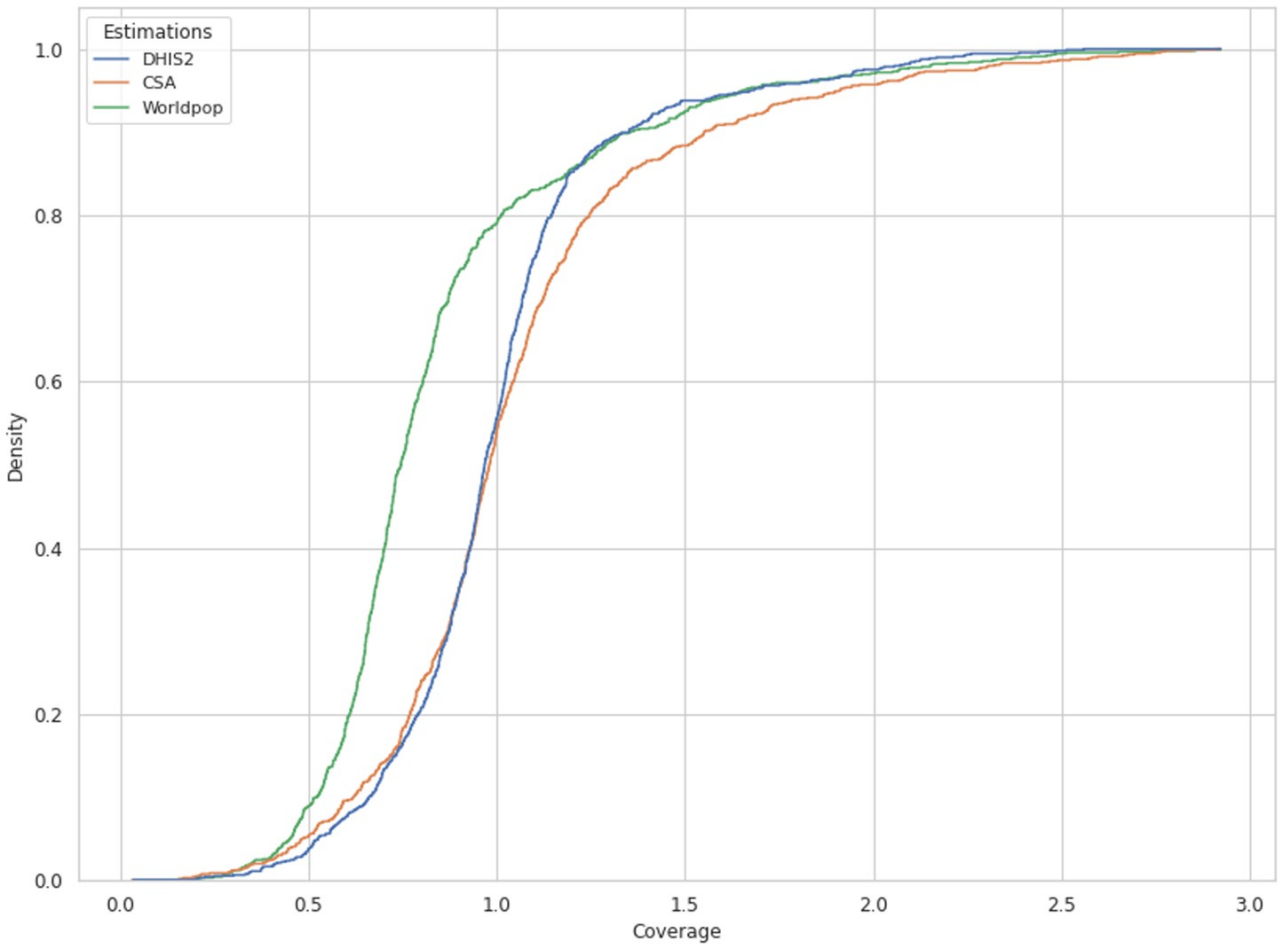
Cumulative distribution comparisons between the three unadjusted administrative coverage estimations for the third dose of diphtheria-pertussis-tetanus-Hib-HepB vaccine (Penta3). Notes: the three unadjusted administrative estimations relied on the target populations of either the WorldPop project, the Central Statistical Agency (CSA), or the District Health Information Software platform (DHIS2). This figure shows how the three estimations depart from each other.

**Fig 2:**
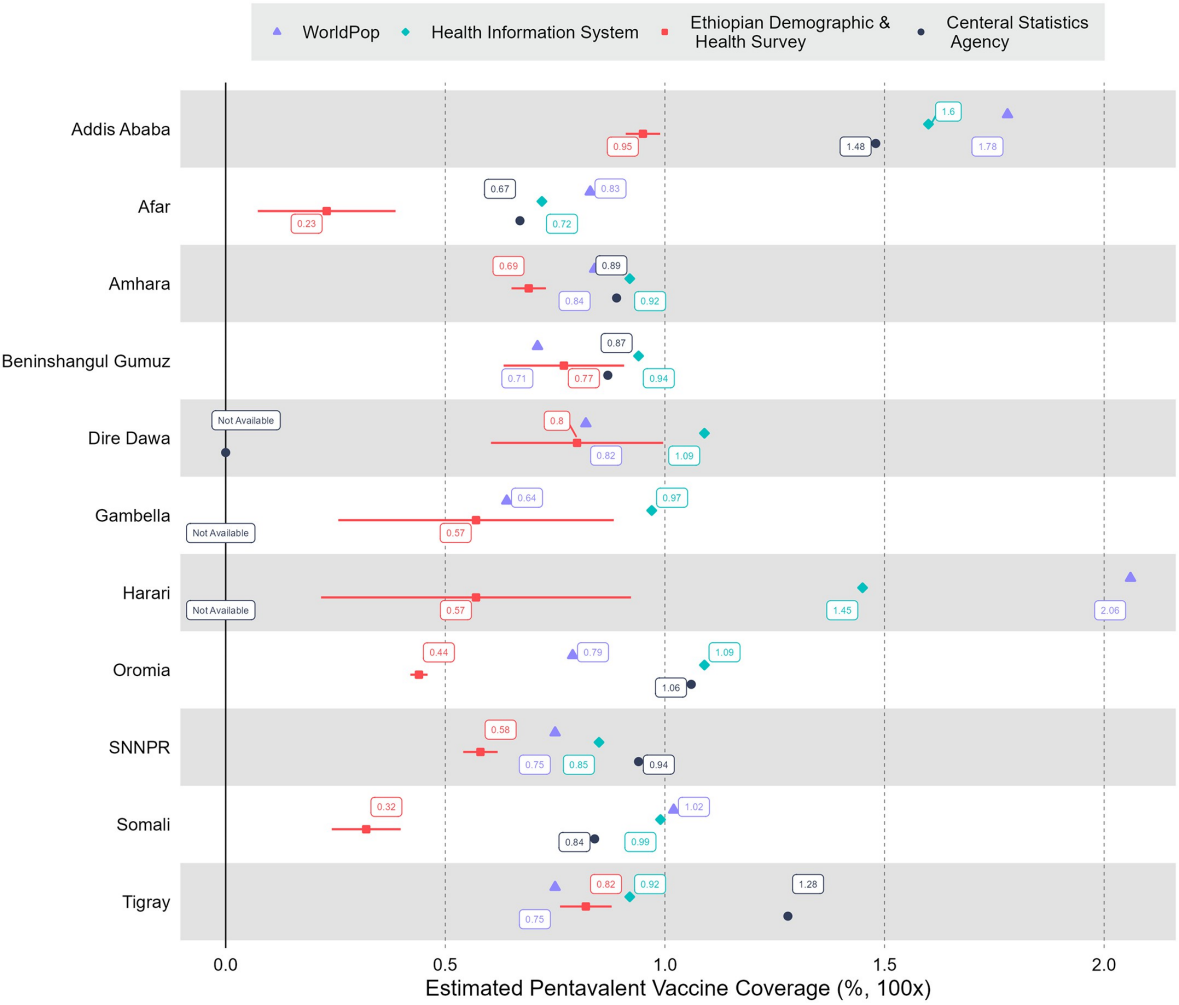
Summary of unadjusted immunization coverages from the WorldPop project (WP), the District Health Information Software platform (DHIS2), and the Central Statistical Agency (CSA), compared with Demographic and Health Survey estimates (EDHS, with 95% confidence intervals). Third dose of diphtheria-pertussis-tetanus-Hib-HepB vaccine (Penta3). SNNPR: Southern Nations, Nationalities and Peoples Region.

**Fig 3:**
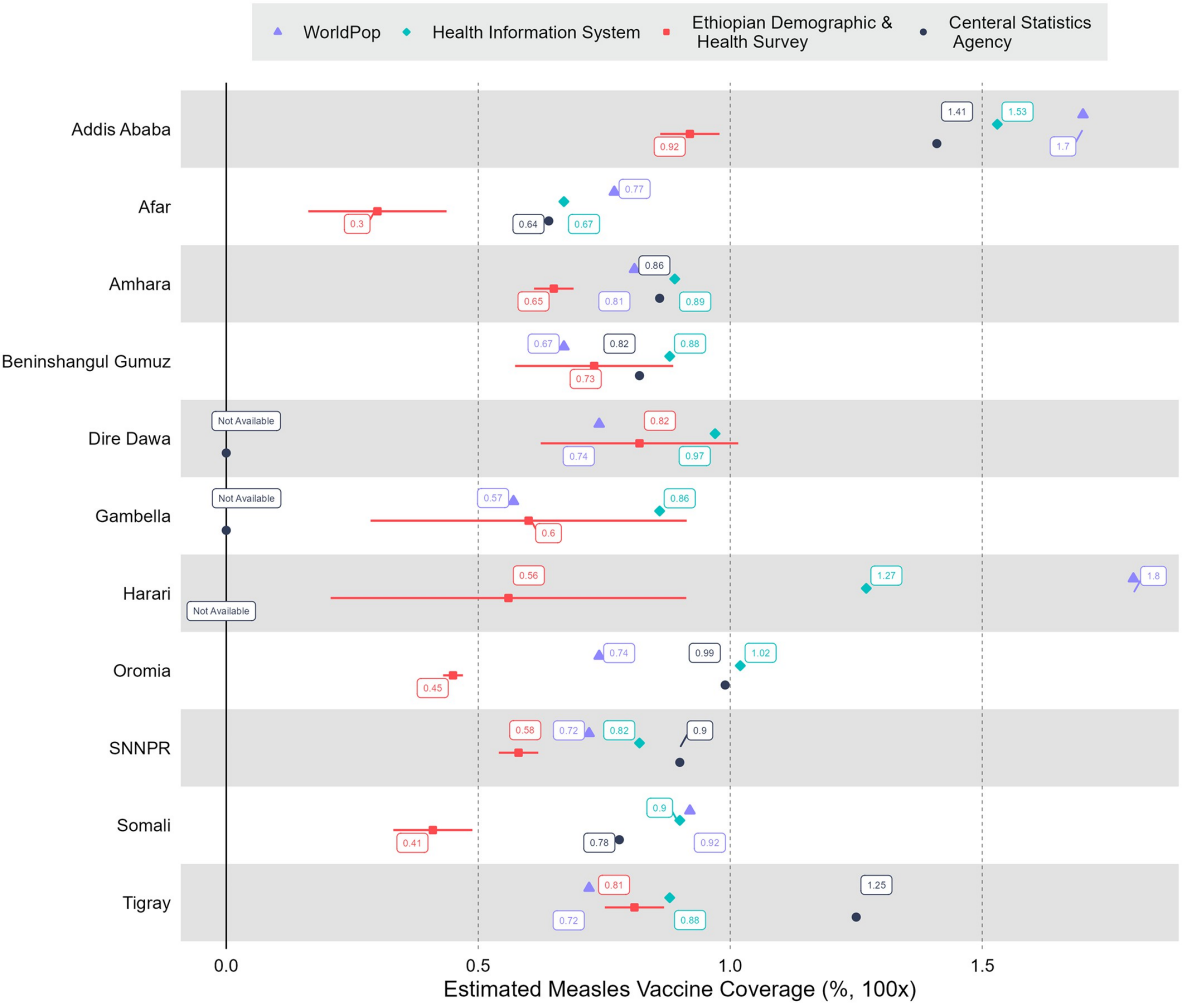
Summary of unadjusted immunization coverages from the WorldPop project (WP), the District Health Information Software platform (DHIS), and the Central Statistical Agency (CSA), compared with Demographic and Health Survey estimates (EDHS, with 95% confidence intervals). First dose of measles vaccine (MCV1), by Ethiopian region. SNNPR: Southern Nations, Nationalities and Peoples Region.

With matched boundary information for WorldPop data along with DHIS2 numerators and denominators, we could estimate coverage for 1,081 woredas; and after removing woredas with extreme coverage values, MCV1/Penta3 coverage was estimated for 1,031 woredas. 477 and 339 woredas had coverage greater than 100% for Penta3 and MCV1, respectively. The unadjusted weighted average coverage for Penta3 and MCV1 were 99% and 92%; with coverage significantly greater than 100% for most woredas. The estimation using DHIS2 numerators and CSA denominators (after CSA is scaled to match the WorldPop data) consisted of 901 woredas: 420 and 336 woredas had coverage greater than 100% for Penta3 and MCV1. For the CSA denominator, the unadjusted coverage for Penta3 and MCV1 was 99% and 94%. For the WorldPop denominator, 261 and 238 woredas had coverage greater than 100% for Penta3 and MCV1, respectively. The number of woredas with more than 100% coverage when using WorldPop denominators was less than for DHIS2 and CSA denominators (WorldPop had on average larger populations). Fig 1 shows the cumulative distribution comparisons between unadjusted administrative estimations: CSA and DHIS2 estimations had coverage close to each other and an almost similar percentage of estimates below 100%. According to EDHS 2019, the percentage of children immunized for Penta3 and MCV1 was 61% and 59%, respectively [5]. Comparing these two estimates to the weighted mean of the three administrative coverages, there were significant variations. The WorldPop unadjusted national estimation was close to EDHS compared to that of CSA and DHIS2 estimations. Figs 2 and 3 show the weighted average of the unadjusted estimations compared to EDHS coverage. As shown, the EDHS estimation for Harari and Gambella had a wide confidence interval due to the small sample size, despite integration of 2019 and 2016 EDHS. The variations in CSA, DHIS2, and WorldPop estimations and that of EDHS were substantial, across a specific region.

After adjustments, all coverage estimates were bounded within 0 and 1 (0–100%). Fig A6.16–17 (S6 Text) show scatter plots of adjusted vs. unadjusted estimations for DHIS2 and WorldPop denominators for Penta3. For high/low unadjusted coverage, we expected the adjustment model to generate high/low adjusted coverage as well. However, the level of adjustment for different regions depended on the values of EDHS regions used for calculating the variance of the theoretical distribution.

**Fig 4:**
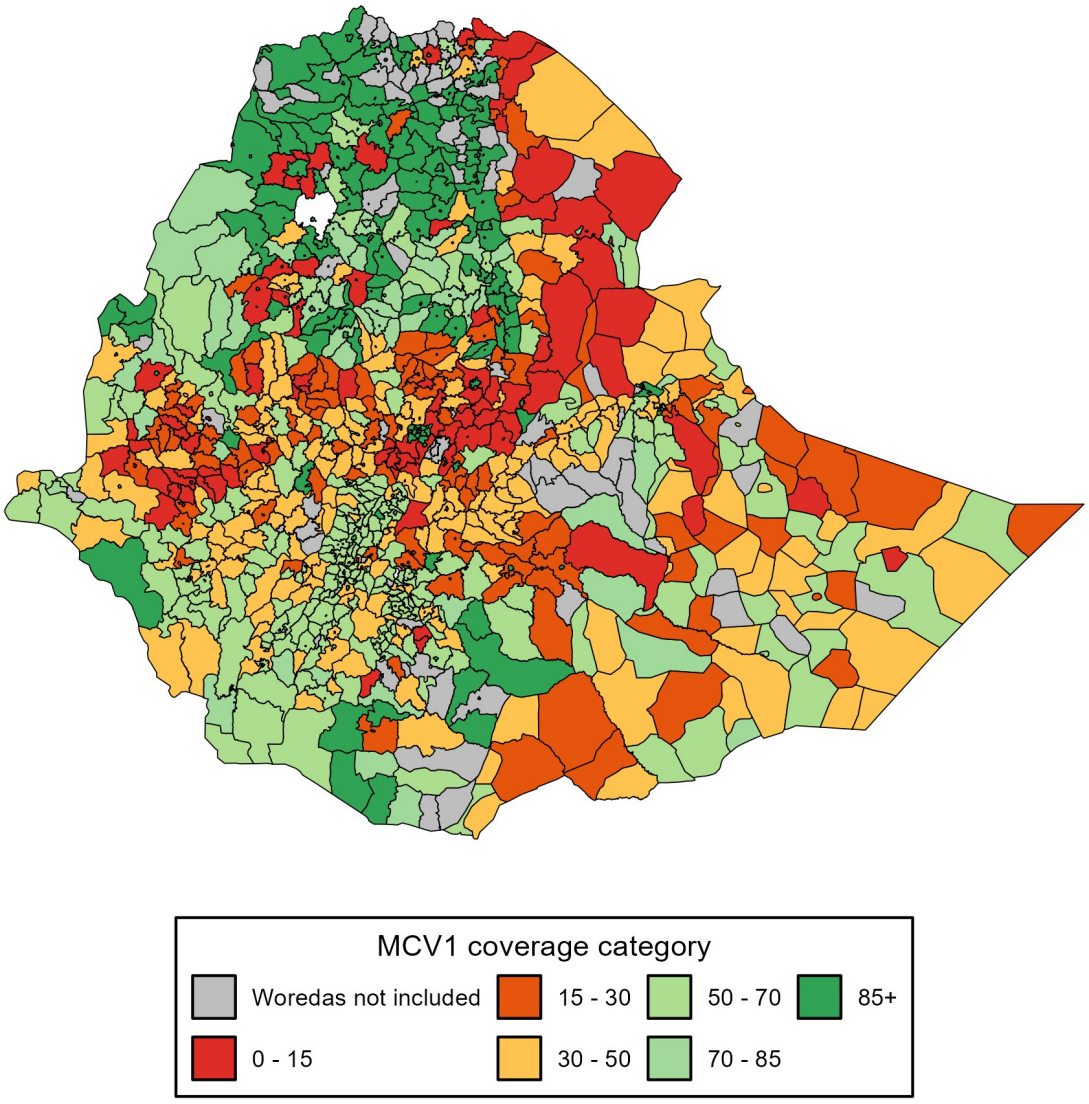
Immunization coverage estimates at the Ethiopian woreda level. First dose of measles vaccine (MCV1). Note: This figure shows results for 1031 woredas.

**Fig 5:**
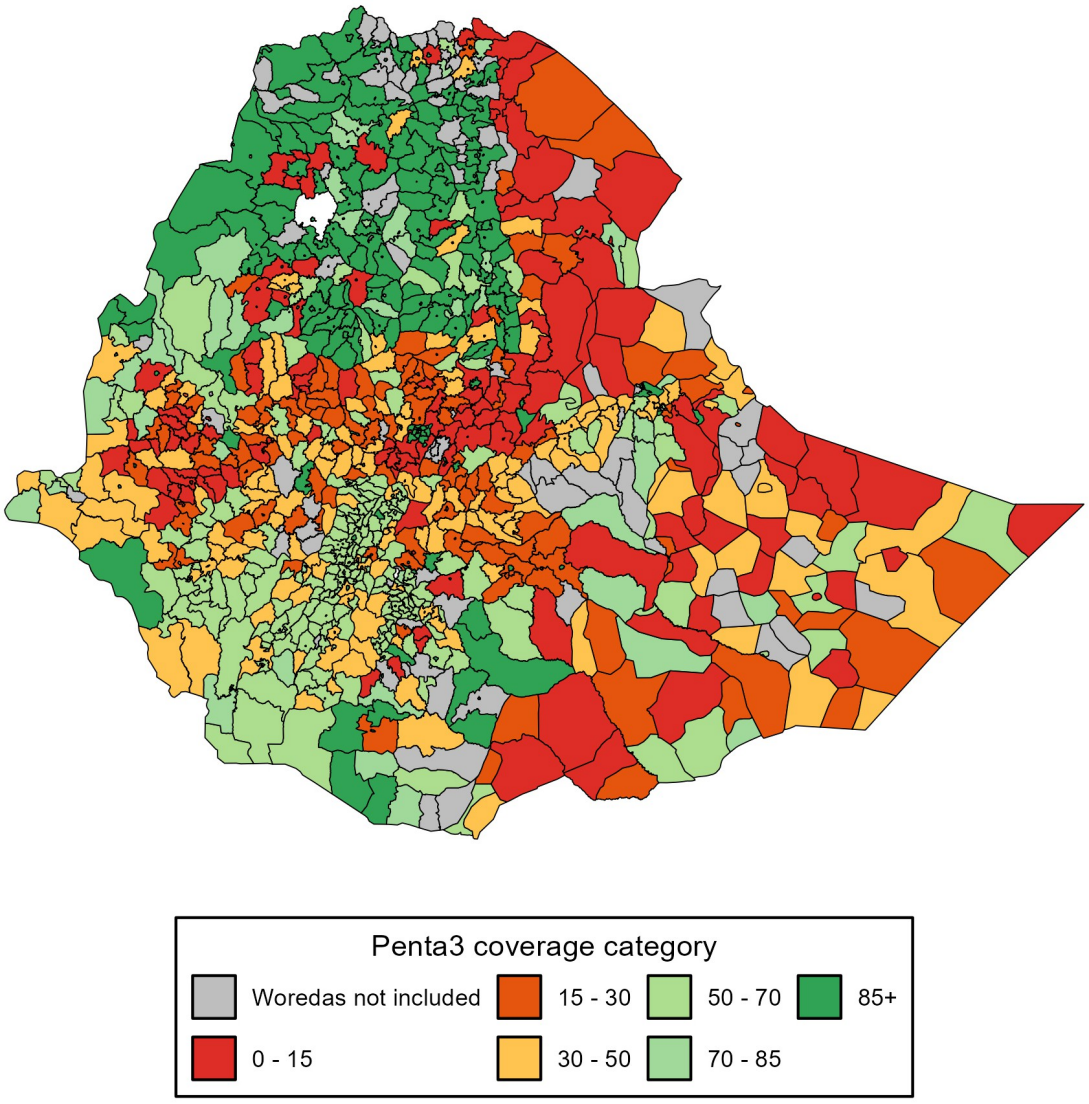
Immunization coverage estimates at the Ethiopian woreda level. Third dose of diphtheria-pertussis-tetanus-Hib-HepB vaccine (Penta3). Note: This figure shows results for 1031 woredas.

For woredas in some regions, the correction model might perform poorly. For instance, for 29 Somali woredas, the correction provided underestimated values for WorldPop. However, since we also used DHIS2 and CSA, we could generate more triangulated estimations. For woredas in which the corrected coverage was poor across all three denominators, they would be labelled as unreliable; however, these uncertainties were also due to EDHS. Figs 4 and 5 show the final adjusted coverage for Penta3 and MCV1 across woredas. Addis Ababa and Dire Dawa city administrations, and Tigray had the highest coverage levels. For Addis Ababa, MCV1 coverage ranged between 83 and 100%, and Penta3 from 88 to 100%, across its 11 sub-cities. For Dire Dawa, MCV1 coverage ranged from 56 to 100%, and Penta3 from 61 to 100%, across its 9 sub-cities. In Tigray, MCV1 coverage ranged from 8 to 100%, whereas, for Penta3 it ranged from 4 to 100%, across its 47 woredas.

**Fig 6:**
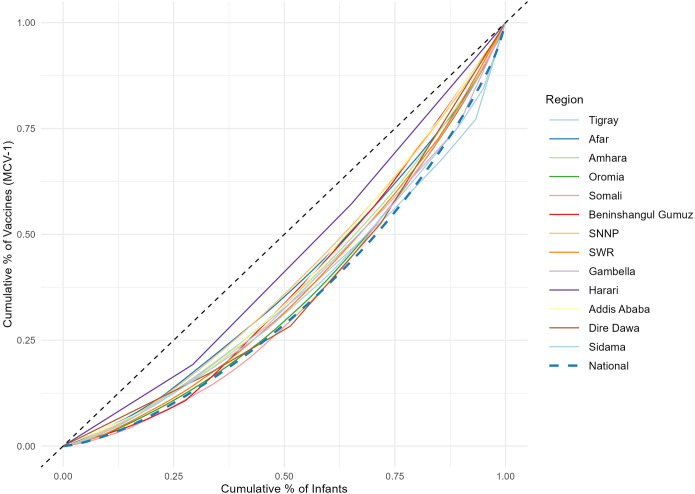
Lorenz curves for assessing disparities in immunization across woredas within Ethiopian regions. Disparities in the first dose of measles vaccine (MCV1). Notes: The y-axis shows the cumulative percentage of vaccines administered for each woreda, whereas the x-axis shows the cumulative percentage of the target population. SNNPR: Southern Nations, Nationalities and Peoples Region.

**Fig 7:**
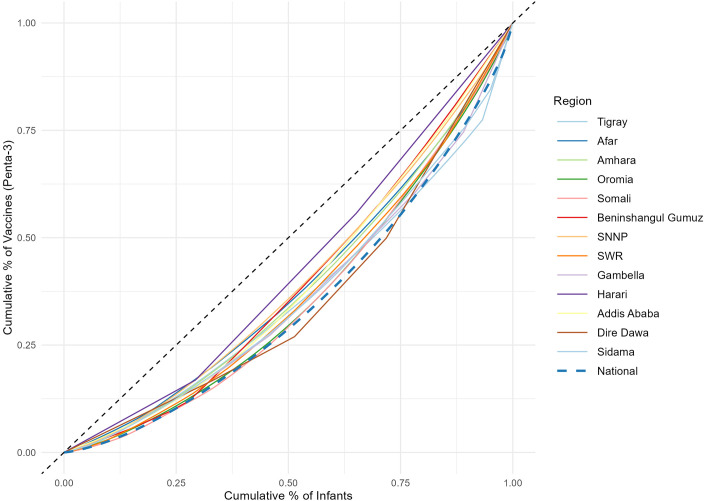
Lorenz curves for assessing disparities in immunization across woredas within Ethiopian regions. Disparities in the third dose of diphtheria-pertussis-tetanus-Hib-HepB (Penta3) vaccine. Notes: The y-axis shows the cumulative percentage of vaccines administered for each woreda, whereas the x-axis shows the cumulative percentage of the target population. SNNPR: Southern Nations, Nationalities and Peoples Region.

In assessing disparities across woredas, we estimated Lorenz curves with continuous distribution functions and Gini indices (S8 Text) (Figs 6 and 7). At the national level, the Gini index for Penta3 was 0.36, and 0.37 for MCV1. However, there were great variations across woredas within regions. For example, for Somali, the Gini for Penta3 was 0.36, and 0.37 for MCV1. In addition to great heterogeneity, Somali had also low coverage levels. The Gini index for Harari was only 0.16 for Penta3 and 0.13 for MCV1, indicating much lower disparity levels. Afar is one of the regions with the lowest coverage for Penta3/MCV1, and its Gini index was 0.23 for both Penta3 and MCV1 (Table 2; S8 Text). The disparities in Penta3 and MCV1 were very substantial across woredas in the country (Figs 4 and 5). The correlation for MCV1 and Penta3 Gini indices was very high (0.98), which points that either immunization indicator would capture the same disparity diagnosis.

**Table 2:** Gini index for the third dose of diphtheria-pertussis-tetanus-Hib-HepB (Penta3) and first dose of measles (MCV1) immunizations, by Ethiopian region.

Region	Gini coefficient-Penta3	Gini coefficient-MCV1
Addis Ababa	0.23	0.23
Afar	0.23	0.23
Amhara	0.27	0.28
Harari	0.16	0.13
Gambella	0.28	0.28
Benishangul Gumuz	0.28	0.30
Sidama	0.30	0.30
Dire Dawa	0.28	0.27
Tigray	0.28	0.28
SNNPR	0.27	0.27
Oromia	0.33	0.33
South West	0.32	0.32
Somali	0.36	0.37
National	0.36	0.37

Notes: The Gini index ranges from 0 to 1, with 0 indicating perfect equality and 1 indicating perfect inequality. The underlying Lorenz curve was assumed to follow a beta distribution. SNNPR: Southern Nations, Nationalities and Peoples Region.

## Discussion

The use of health management information system data, such as DHIS2, for generating annual and district-level estimations of health services coverage is crucial in low- and middle-income countries, especially when triangulated with large household surveys, in the context of tracking progress toward national objectives and international goals. In this paper, we presented methods for addressing data challenges emanating from DHIS2 and used the DHS for adjusting the observed overestimations in administrative coverage often due to uncertainties in the census data. This paper attempts to provide guidance on utilizing DHIS2 data of moderate quality. This is especially relevant for countries with less frequent yet costly household surveys and facing multifaceted data collection challenges.

We provide information on the recent Penta3 and MCV1 coverage in Ethiopian woredas. Ethiopia’s second Health Sector Transformation Plan (HSTP-II) had targets of 85% Penta3 coverage by 2025 (from 61% in 2019) [2]. Similarly, the SDGs set universal coverage targets by 2030. To effectively implement national immunization plans, vaccine coverage estimates at the district level are a necessity. In Somali for instance, Penta3 coverage ranged from less than 5% to around 85% across woredas; even in urban areas where relatively disparities are lower, like Addis Ababa, the smallest MCV1 coverage (83%) was far lower than the 100% coverage estimates for several other sub-city woredas. Overall, we found that MCV1 coverage in many woredas was far lower than 95%, the threshold indicated for measles elimination goals [32]: 87% of woredas presented MCV1 coverage below 95%, 54% of them being in Oromia. Active measles outbreaks have been ongoing in the country since January 2022 and as of August 2022 around 4,000 confirmed cases had been reported [33, 34]. The most common risk factor for these outbreaks was the large percentage of unvaccinated children and the interruption of routine immunization services. As for Penta3, a 72% coverage of intermediate target for 2022 was indicated by HSTP-II [2]. However, we estimated a large number of woredas with low coverage in the country. Future research must then study the barriers to immunizations [29, 35] while taking into account the dynamic socioeconomic determinants of the communities in the relevant woredas.

The Ethiopian Expanded Programme on Immunization implements both outreach community-based campaigns and facility-based immunizations [36]. Vaccine coverage presents stark disparities in Ethiopia, as depicted by the large Gini indeces estimated. These actual disparities could even be further heightened by differences in quality of services observed across woredas in the country. Several studies have pointed to a wide range of health services coverage and quality indices in Ethiopia with regard to gender, geography, and residence [37–39].

Disparities across woredas within the same region were assessed. We found that Harari had the most equitable distribution, while the Somali region had the most unequal one. In Addis Ababa, coverage was high, population is homogeneously urban, and access is less of an issue as shown by the low Gini index. Regions like Somali are more heterogeneous, covering a large geographical area with both urbanized and pastoralist communities, which lead to possibly large inequalities across woredas within the region [40]. The Afar region, which is predominantly pastoral, is presented with lower disparities, as most woredas in the region had lower coverage. Lastly, we note that the number of reported vaccines for both MCV1 and Penta3 increased from 2019 to 2020 (p<0.01), but decreased from 2019 to 2021 (p<0.01). The onset of the COVID-19 pandemic in 2020 and the 2021 internal armed conflicts have challenged the resilience of the Ethiopian health system.

Overall, DHIS2 data were found to be of moderate quality with large variations among regions. For instance, Somali had a high percentage of poor-quality data. The continued updates of the DHIS2 system have shown the commitment of the Ethiopian MoH to increase adherence to HMIS at all levels: we observed increases in the reporting rates over the years since DHIS2 was launched in Ethiopia. Implementing mandatory data reporting guidelines of both public and private health facilities will increase reporting rates and data quality in the future. Ensuring HMIS and DHIS2 are interoperable with existing disease surveillance systems is critical to generate high-quality evidence.

Although prior studies have estimated vaccine coverage at a granular level, for example for measles vaccine using small area estimation methods [41], these efforts typically relied on household surveys and other data sources, rather than HMIS or DHIS2. Our study is novel in blending multiple datasets including DHIS2, DHS, and various census population data to generate vaccine coverage estimates at the district level in Ethiopia. To our knowledge, it is one of the first studies that attempts to correct biases in target population groups due to inaccurate census estimates. For most low- and middle-income countries, due to a lack of frequent census, population numbers are often underestimated and national immunization coverage surveys are often rapidly out of date (Ethiopia last implemented such a survey in 2012 [42]). The preliminary methods we expose here could be adapted for many other low- and middle-income countries also utilizing the DHIS2 platform.

Nevertheless, our study presents a number of limitations. First, ideal datasets for benchmarking would be immunization-specific surveys; however, in Ethiopia, such data are not available, and as an alternative, we used EDHS data. The most recent EDHS (2019) was an interim DHS and we had to increase its sample size by integrating 2016 and 2019 EDHS. Given that DHS are cross-sectional, we had to ignore temporal variation. The impact of this assumption would vary on how a given variable representing a certain service or outcome changes over time. Second, in calculating the variance of coverage across woredas we at times had small sample sizes at the woreda level. The sample of woredas used in variance calculations was drawn from samples of varying sizes of individuals by woreda. Each woreda was given the same weight, and this might explain the high inter-woreda variances in our coverage estimates. Third, no efforts were made to identify possible sources of outliers for the DHIS2 headcounts. Sometimes outliers might occur due to data entry, reporting, or processing errors. However, in some cases, there might be reasons for sudden decreases or increases in utilization. For instance, the decrease in the number of vaccines administered in Afar or Tigray regions after November 2021 would likely be due to local armed conflicts; in Addis Ababa for a few months, we also observed decreases in vaccines provided during the COVID-19 pandemic. Fourth, despite our best effort to correct for DHIS2 data, additional efforts are required to address data quality gaps at the level of reporting facilities. Fifth, events like migration (e.g., relocation, constant mobility) which could be related to different factors, might result in either overestimation or underestimation of coverage due to varying target population sizes.

Both routine outreach services and campaign-based delivery play significant roles. Routine outreach services ensure consistent and regular access to vaccines, particularly in rural and remote areas. These services operate all year-round. Data for these services are routinely captured in DHIS2 and contribute to regular coverage estimations. Campaign-based immunizations, on the other hand, are typically conducted sporadically to target specific diseases or to rapidly increase coverage in response to outbreaks. While these campaigns can greatly enhance coverage rates over a short time-period, they do not reflect typical immunization coverage levels and are not commonly reported. To better capture administrative immunization coverage, it would be beneficial to track data from both routine outreach services and campaign-based delivery, separately. This would provide a nuanced understanding of coverage rates and of the distinct impact of these two delivery strategies. Efforts should also be made to ensure complete and timely reporting of all immunization activities to improve DHIS2 data quality.

National and regional planning can leverage local coverage estimates to reduce vaccine coverage disparities across districts to attain national and international goals. Future work should focus on refining such district-level vaccine coverage estimations and on identifying the constraints to design effective programs for advancing toward universal health coverage.

## Supporting information

S1 Text Definitions for the third dose of pentavalent (Penta3) and the first dose of measles vaccine (MCV1). (PDF)

S2 Text Target population and EDHS datasets. (PDF)

S3 Text DHIS2 data management. (PDF)

S4 Text DHIS2 and WorldPop woreda matching—Levenshtein distance. (PDF)

S5 Text Summarizing the corrected estimations for the three denominators. (PDF)

S6 Text Results from data management methods and correction model. (PDF)

S7 Text Correcting administrative coverage with EDHS estimates. (PDF)

S8 Text Immunization disparities across woredas in Ethiopia. (PDF)
