# Supplementary material for: Estimating immunization coverage at the district level: A case study of measles and diphtheria-pertussis-tetanus-Hib-HepB vaccines in Ethiopia
Source: PLOS Glob Public Health. 2024 Jul 25;4(7):e0003404. doi: 10.1371/journal.pgph.0003404 (PMC11271922; doi:10.1371/journal.pgph.0003404)
Supplement: S1 Text — (PDF) [file pgph.0003404.s001.pdf]

S1 Text: Definitions for the third dose of pentavalent (Penta3) and the first dose of measles vaccine (MCV1)

For MCV1, the "numerator" was chosen to be the number of children under one year of age who had received at least one dose of measles vaccine in a given year.

For Penta3, the numerator was chosen to be the number of children under one year of age who had received the three doses of pentavalent DTP-Hib-HepB vaccine in a given year.

For the target population or "denominator", the total number of individuals would be, for both MCV1 and Penta3, the total number of infants surviving to age one in a given year.
