# Supplementary material for: Estimating immunization coverage at the district level: A case study of measles and diphtheria-pertussis-tetanus-Hib-HepB vaccines in Ethiopia
Source: PLOS Glob Public Health. 2024 Jul 25;4(7):e0003404. doi: 10.1371/journal.pgph.0003404 (PMC11271922; doi:10.1371/journal.pgph.0003404)
Supplement: S2 Text — (PDF) [file pgph.0003404.s002.pdf]

S2 Text: Target population and EDHS datasets

The denominators are collected from multiple data sources to extract the required target populations. To increase the sample size in survey data, EDHS 2016 and 2019 were integrated.

Table A2.1. Conversion factors to obtain the total number of surviving infants from Central Statistical Agency (CSA) population data.

Location	Conversion factor
National	0.0316
Tigray	0.0322
Afar	0.0269
Amhara	0.0311
Oromia	0.0322
Somali	0.0294
Benishangul Gumuz	0.0307
South Nations Nationalities and Peoples Region	0.0319
Gambella	0.0277
Harari	0.0289
Dire Dawa	0.0303
Addis Ababa	0.0224

Areal interpolation: Areal interpolation is a technique that consists of recalculating data defined over a set of regions when the boundaries between these regions change. It is a multi-step procedure. First, the original boundaries are overlaid with the new ones. This defines a set of sub-regions, which are subdivisions of both the original and the new regions. We calculate the areas of each sub-region, which we divide by the area of the original region. This gives a percentage for each sub-region. We then multiply by the value of our variable in the original region to get the values in the sub-regions. Finally, we sum the values of all sub-regions in each region.

This method was used to estimate the population of CSA woredas on WorldPop boundary data. 33 (mostly town woredas) had highly underestimated population values. This was because the Bureau of Finance and Economic Development (BoFED) woreda boundary data mostly considered town and rural parts of a woreda separately. However, the CSA merged town and rural parts of some woredas into one big woreda. This created a scenario where a CSA woreda corresponded to multiple BoFED (or WorldPop) woredas. In most cases, this caused a boundary mismatch between the two administrative structures. For all 33 town woredas with associated rural woredas in DHIS2, the population ratio of rural to town woreda was used as an adjusting factor for these woredas.

WorldPop and BoFED data: The WorldPop project data are available in two types, constrained and unconstrained. The unconstrained population data are generated by estimating the population across all land-based grid cells on a global scale. However, in this paper, constrained population data was used: this is because population estimation was carried out for the areas that have built settlements [1]. The WorldPop data was adjusted to match the corresponding United Nations population estimates. This data was generated by the application of Random Forest models developed by Stevens [2]. The spatial resolution of the grid square population data was 100m by 100m. In the case of Ethiopia, the last census was conducted in 2007. Thus, estimation of population data from different sources was used. However, for this paper, WorldPop gridded data was used, and also the population count was extracted using zonal statistical tools at the woreda level. One advantage of using WorldPop data is that the estimation depends on multiple covariates including vegetation cover, built settlements, roads, health facilities, satellite images, and others. The accuracy of the population distribution estimation is highly dependent on the resolution of the satellite image where the built settlement is mapped. The population estimation is more accurate where buildings are accurately mapped. Hence, it is likely to have a very small population estimate in uninhabited areas and an exaggerated population estimate in densely built-up areas. Despite these limitations, where up-to-date census is not available, WorldPop data is believed to give good population distribution data. The WorldPop data is then overlaid with updated boundary administration to extract the population at regional, zonal, and woreda levels.

DHIS2 denominator: This denominator was extracted from DHIS2. The data were extracted for the years 2017/2018, 2019, 2020, and 2021/2022. However, for some woredas, a data point might be missing for some years. If we had 2+ data points, the population for the remaining year(s) was estimated by assuming exponential population growth. If only one data point was available, the growth rate was calculated at the regional level and used to estimate the remaining missing data points for the woreda. If there was no data available for all the years, then the woreda was removed for this specific denominator depending on the value of its numerator. This means that if the woreda contained a significant number of reported vaccines, for both MCV1 and Penta3, then the denominator would be borrowed from CSA or WorldPop, depending on the availability of the woreda within these two denominators. The CSA denominator was available for 2020 and 2021, whereas for WorldPop 2020, depending on where the data was borrowed the above approach would be used to estimate the population for the remaining years. There are frequent administrative boundary changes in Ethiopia, across regions, zones, and woredas. For instance, Sidama became an independent region from SNNPR in 2020. Similarly, in 2022, SWR became separated from SNNPR. Usually, such kinds of changes are also captured and updated for all the indicators across the years within DHIS2, i.e. before and after the change. However, for the denominator, DHIS2 would only update the data for years after the change happened. The population data for the years before the change would remain unchanged. Accordingly, for woredas where such kinds of changes were observed, usually, the population data seemed to decrease, as a portion of the population would be moved to the new structure after the change. For these woredas, we corrected data points before the change proportionally to that of data values after the change by using the other two denominators and the above approach.

Grouping of health facilities: In DHIS2, around 450 health facilities, mostly hospitals were not categorized into their respective areas (woredas). Of these health facilities, 397 facilities, with considerable data points were grouped. For this, two approaches were developed:

- The health facilities were geocoded by using Google maps and other local health facility data sources like Master Facility Registry (MFR), Cold chain, Health Resource Availability Monitoring System (HeRAMS), and different datasets stored at the National Research Tracking Database Management

system. Then, from latitude and longitude, we performed reverse geo-coding to identify the respective woredas, zones, and regions.

- Some health facilities did not match with the above method. In this case, semantic analyses were performed. This process took the name of the health facility and looked for woredas with the same name (at least containing similar words). If exactly one match was found, it was grouped automatically. If more than two matchings were found, we did manual grouping.

However, for a few health facilities (n=53) we could not find the respective areas (woredas), thus we removed them from our analysis.

EDHS data integration: In our study, the DHS was designed to yield representative data at the regional and national levels. However, at the woreda (district) level, the sample size became a limiting factor, preventing the generation of representative coverage estimates. This limitation, coupled with the temporal gap between our administrative coverage data and the DHS year, largely explained the poor correlation between our adjusted administrative coverage and DHS estimates at the district level.

Given these discrepancies, we could not anticipate a direct correspondence between unadjusted administrative coverage at the woreda level and DHS estimates. To leverage the DHS as a benchmark, our model met two criteria:

1. Not to be informed by the DHS district-level coverage estimates.
2. To be informed by the national and regional coverage estimates from the DHS.

With these considerations in mind, we employed a percentile matching technique, aligning administrative coverage with a hypothetical beta distribution which was parameterized using expectation and variance derived from the DHS.

With the 2019 Mini DHS, estimating district level coverage was challenging. Therefore, we have decided to integrate it with the 2016 DHS. To do that, first, for each survey data we merged the vaccine record from Children's Recode (KR) data with the geographic data using cluster IDs. This added cluster geographic information to KR datasets for both years. We then concatenated the 2016 and 2019 datasets, giving us a single dataset with the clusters from both years. Finally, we identified the districts in which the clusters were located and derived their respective immunization coverage.

Figure A2.1 shows the extent to which the respondents within the cluster were representative depending on responding women's sample weight. The darker and narrower the circle the smaller the sample size within that cluster, whereas the yellow and the bigger the circle the larger the sample size.

Figure A2.1. Cluster locations of EDHS after merging of the EDHS 2016 and EDHS 2019 surveys.

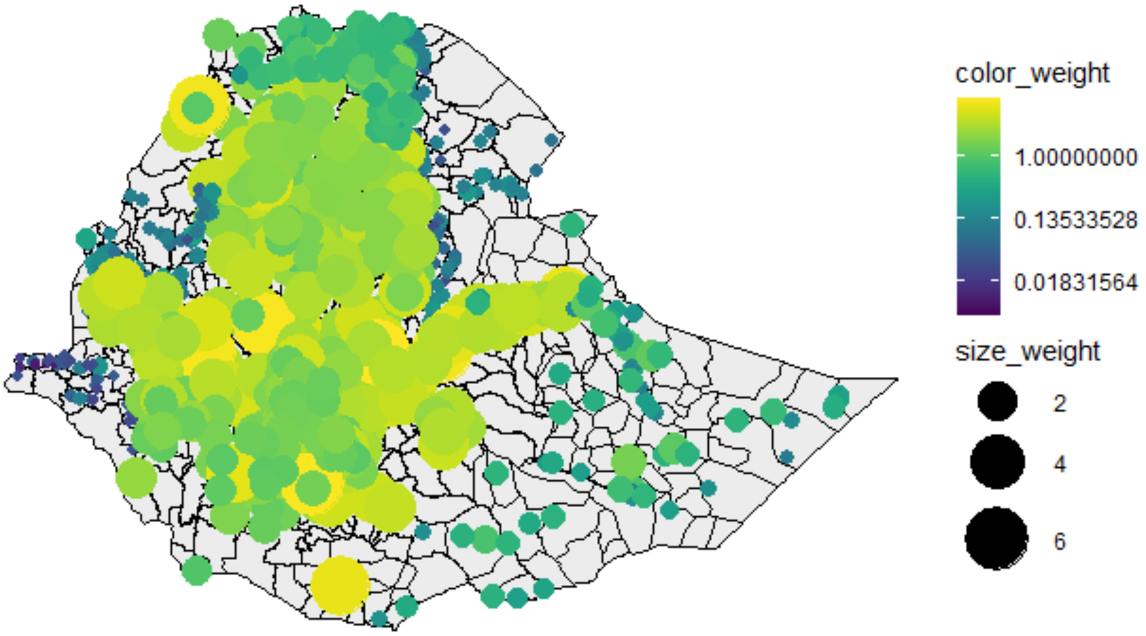

References

1. Top-down estimation modelling: Constrained vs Unconstrained - WorldPop [Internet]. [cited 2022 Aug 21]. Available from: https://www.WorldPop.org/methods/top_down_constrained_vs_unconstrained/
2. Stevens FR, Gaughan AE, Linard C, Tatem AJ. Disaggregating census data for population mapping using Random forests with remotely-sensed and ancillary data. PLOS One 2015;10(2):1-22.
