# Supplementary material for: Estimating immunization coverage at the district level: A case study of measles and diphtheria-pertussis-tetanus-Hib-HepB vaccines in Ethiopia
Source: PLOS Glob Public Health. 2024 Jul 25;4(7):e0003404. doi: 10.1371/journal.pgph.0003404 (PMC11271922; doi:10.1371/journal.pgph.0003404)
Supplement: S3 Text — (PDF) [file pgph.0003404.s003.pdf]

S3 Text: DHIS2 data management

Missing value handling: There are multiple methods for handling missing values for numeric data such as using means and linear regressions. However, such simple approaches reduce the variance of data after imputation [1]. In this work, we implemented Multiple Imputations by Chained Equation (MICE). MICE imputes missing data points with the most plausible values. These plausible values are derived from a distribution tailored to each missing data point. The basic assumption of MICE is that the missing data are missing at random [1], which means that the likelihood that a value is missing depends solely on the observed values and can be predicted using them. It imputes data on a variable-by-variable basis, that is woreda-by-woreda basis by specifying an imputation model per variable.

Figure A3.1. Description of the imputation process.

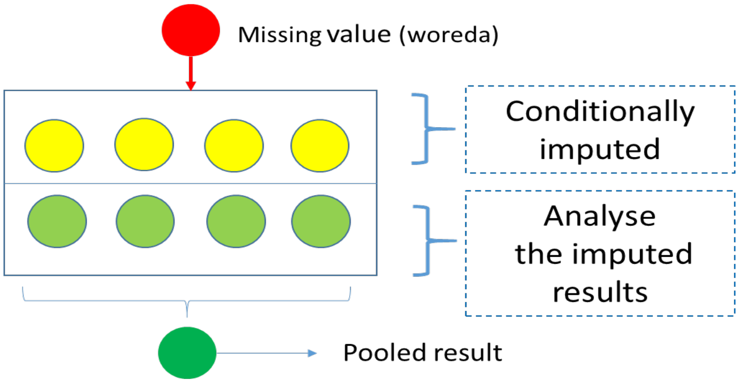

The first step in MICE is to define the number of datasets that need to be imputed. In the example above, four datasets are defined. The non-missing (original) observed data is the same across all imputed datasets, the difference is only in the missing values. Then, the imputed values are analyzed for accuracy and finally, the results for all datasets will be pooled (combining the results from all datasets to generate one imputed value) into one value. The methods used for pooling depends on the data types. As our data is numeric, the mean pooling method was used.

Reference

1. Mera-Gaonaid M, Neumann U, Vargas-Canas R, Ló Pez DM. (2021). *Evaluating the impact of multivariate imputation by MICE in feature selection*. https://doi.org/10.1371/journal.pone.0254720
