# Supplementary material for: Estimating immunization coverage at the district level: A case study of measles and diphtheria-pertussis-tetanus-Hib-HepB vaccines in Ethiopia
Source: PLOS Glob Public Health. 2024 Jul 25;4(7):e0003404. doi: 10.1371/journal.pgph.0003404 (PMC11271922; doi:10.1371/journal.pgph.0003404)
Supplement: S4 Text — (PDF) [file pgph.0003404.s004.pdf]

S4 Text: DHIS2 and WorldPop woreda matching - Levenshtein distance

To obtain estimates of immunization coverage, we need to obtain estimates of the target populations (denominators) for each woreda. First, the denominators provided by DHIS2 have the advantage of matching perfectly with the numerators, so obtaining estimates is straightforward. Our second source of estimates for target populations is WorldPop. The woredas as defined by WorldPop are not always the same as the ones in DHIS2. For instance, the same geographical divisions might have different names in the two datasets; or one woreda in one dataset actually matches several in another dataset. We have 988 woredas given by DHIS2 for 1,081 woredas with WorldPop. The existence of several languages and alphabets in Ethiopia leads to transcriptions in the Latin alphabet with a possible variation of the number of consonants and vowels for the same sound or a modification of the vowel for a close sound. We have one list of woreda names per data source. Our approach is to look, for every DHIS2 woreda, if there is a woreda or group of woredas in WorldPop that correspond. We use the Levenshtein distance to find the woreda of WorldPop that is the most similar to the DHIS woreda. The Levenshtein distance matching is a popular natural language processing approach to match lists of strings. It measures the minimum number of operations to change one word into another. The possible operations are insertion, deletion, or substitution of a single character at a time.

Let us define two lists of strings l_1 and l_2 of respective lengths N_1 and N_2. For every string l_{1i} of l_1 we look for the string l_{2j} in l_2 that minimizes the Levenshtein distance $lev(l_{1i},l_{2j})$. l_{1i} and l_{2j} have respective lengths n_{1i} and n_{2j}. We now compute the Levenshtein distance for every combination (i, j), i=2 1, ..., N_1; j=2 1, ..., N_2 as follows:

$$lev(l_{1i}, l_{2j}) = \begin{cases} 0, & n_{1i} = 0, n_{2j} = 0 \\ n_{1i}, & n_{2j} = 0 \\ n_{2j}, & n_{1i} = 0 \\ Treat(l_{1i}, l_{2j}), & n_{1i} > 0, n_{2j} > 0 \end{cases} .$$

with the *Treat* function defined as:

$$Treat(l_{1i}, l_{2j}) = \begin{cases} lev(pop(l_{1i}), pop(l_{2j})), & l_{1i}[1] = l_{2j}[1] \\ 1 + min \begin{Bmatrix} lev(pop(l_{1i}), l_{2j}) \\ lev(l_{1i}, pop(l_{2j})) \\ lev(pop(l_{1i}), pop(l_{2j})) \end{Bmatrix}, & l_{1i}[1] \neq l_{2j}[1] \end{cases}$$

The pop function takes a string as argument and removes its last character. We apply this to our two lists of woreda names and obtain the following global matrix $LEV(l_1, l_2)$:

$$LEV(l_{DHIS2}, l_{Worldpop}) = LEV(l_1, l_2) = \begin{pmatrix} lev(l_{1,1}, l_{2,1}) & \cdots & lev(l_{1,1}, l_{2,N_2}) \\ \vdots & \ddots & \vdots \\ lev(l_{1,N_1}, l_{2,1}) & \cdots & lev(l_{1,N_1}, l_{2,N_2}) \end{pmatrix}$$

We then pick, for every woreda name in DHIS2, the closest woreda name in WorldPop in terms of Levenshtein distance. This procedure allowed to match most of the woredas correctly. When we could not find any match, which usually means that the data does not exist in the other dataset or that the woredas simply did not match, we ignored them for that dataset and imputed a null value. Further validation was done to make sure all matchings were correct. Using Excel, a manual validation was done for semantic matching. In addition, further validation was done against BoFED boundary data.

One of the problems frequently encountered while estimating vaccine coverage is the fact that the matching is not always one-to-one between DHIS2 numerator and WorldPop denominator. In some cases, a woreda within DHIS2 will correspond in WorldPop to two woredas divided in urban and rural woredas. Accordingly, if several denominator woredas matched a single DHIS2 woreda, then we summed the population of the denominators, which gave us a single denominator. The opposite situation might also occur, in which case, the DHIS2 was merged to create vaccine data for the larger woreda.
