# Supplementary material for: Estimating immunization coverage at the district level: A case study of measles and diphtheria-pertussis-tetanus-Hib-HepB vaccines in Ethiopia
Source: PLOS Glob Public Health. 2024 Jul 25;4(7):e0003404. doi: 10.1371/journal.pgph.0003404 (PMC11271922; doi:10.1371/journal.pgph.0003404)
Supplement: S5 Text — (PDF) [file pgph.0003404.s005.pdf]

S5 Text: Summarizing the corrected estimations for the three denominators

We proceed to: d, DHIS to DHIS estimation; c, DHIS to CSA estimation; and w, DHIS to WorldPop estimation. Then we calculated the absolute percentage differences between each estimation: L1 percentage for d to c; L2 for c to w; and L3 for d to w. Then we defined a threshold of 20% to label a certain estimation as trustworthy or not. For a given woreda, if one of the three corrected estimations was missing, because of a missing denominator, the methods summarized in Table A5.1 would apply to the remaining two coverages.

Table A5.1. Summarizing the coverage results of the three denominators.

Percentage difference values	Summarization method
L1, L2, and L3 > 20%	Unreliable
L1, L2, and L3 < 20%	Mean of c, d, and w
L1 and L2 < 20%, but L3 > 20%	Median of c, d, w, i.e. c
L1 and L3 < 20%, but L2 > 20%	Median of c, d, w, i.e. d
L2 and L3 < 20%, but L1 > 20%	Median of c, d, w, i.e. w
L1 and L2 > 20%, but L3 < 20%	Mean of d and w
L1 and L3 > 20%, but L2 < 20%	Mean of c and w
L2 and L3 > 20%, but L1 < 20%	Mean of d and c
