# Supplementary material for: Estimating immunization coverage at the district level: A case study of measles and diphtheria-pertussis-tetanus-Hib-HepB vaccines in Ethiopia
Source: PLOS Glob Public Health. 2024 Jul 25;4(7):e0003404. doi: 10.1371/journal.pgph.0003404 (PMC11271922; doi:10.1371/journal.pgph.0003404)

S6 Text: Results from data management methods and correction model

Figure A6.1. The first dose of measles (MCV1) vaccine coverage for woredas included in EDHS (2016 and 2019) surveys, before adjustment and estimation.

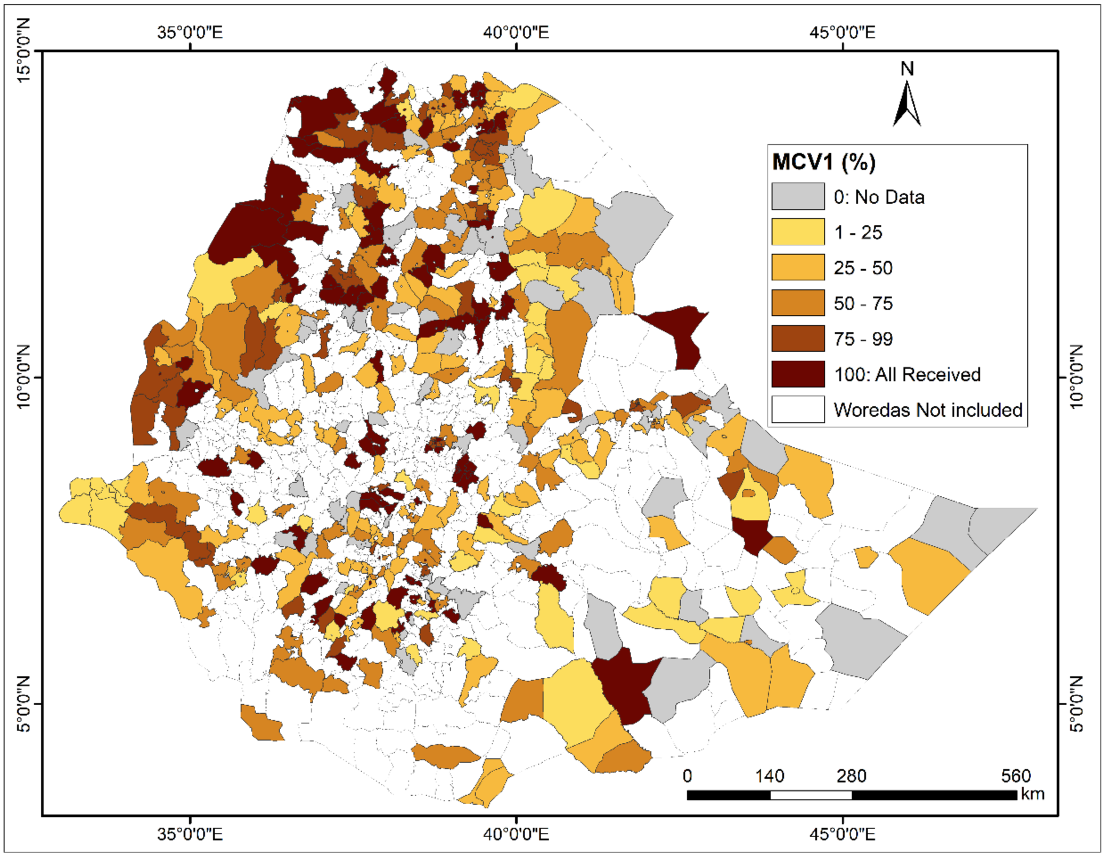

Figure A6.2. The third dose of diphtheria-pertussis-tetanus-Hib-HepB (Penta3) vaccine coverage for woredas included in EDHS (2016 and 2019) surveys, before adjustment and estimation.

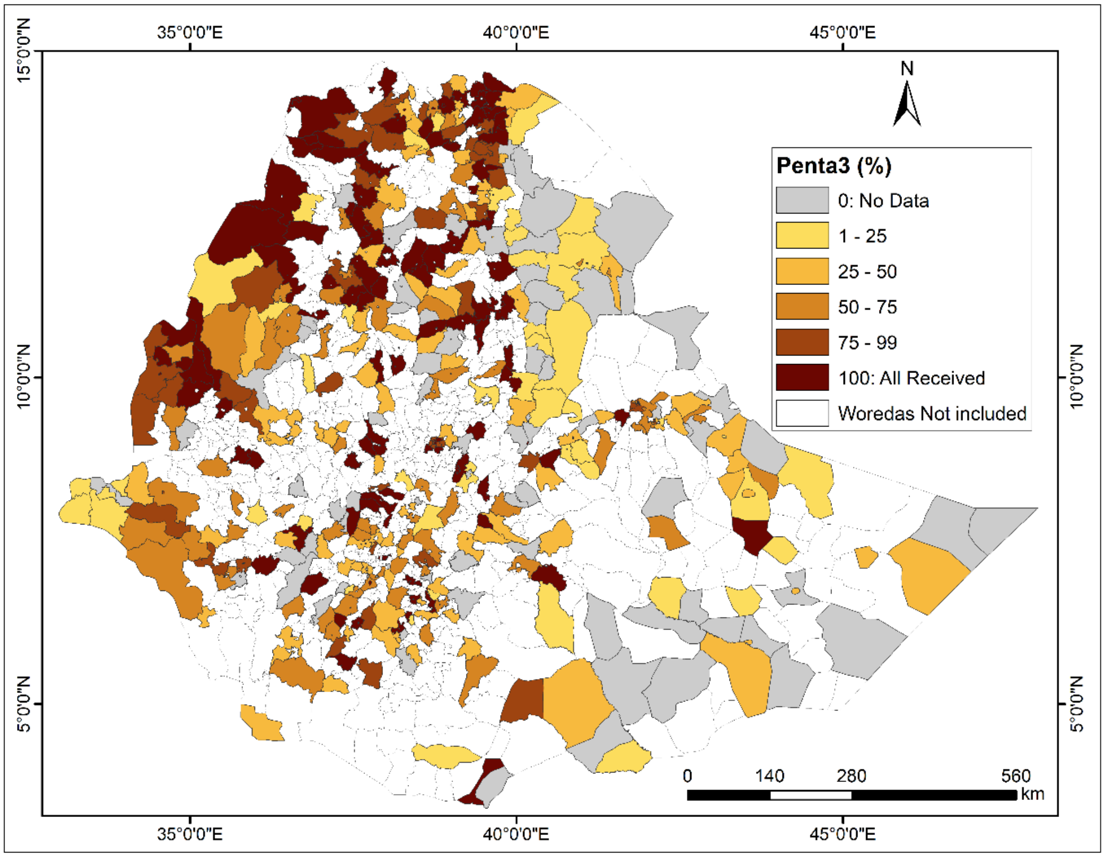

Figure A6.3. The first dose of measles (MCV1) vaccine coverage for regions of Ethiopia from EDHS (2016 and 2019) surveys.

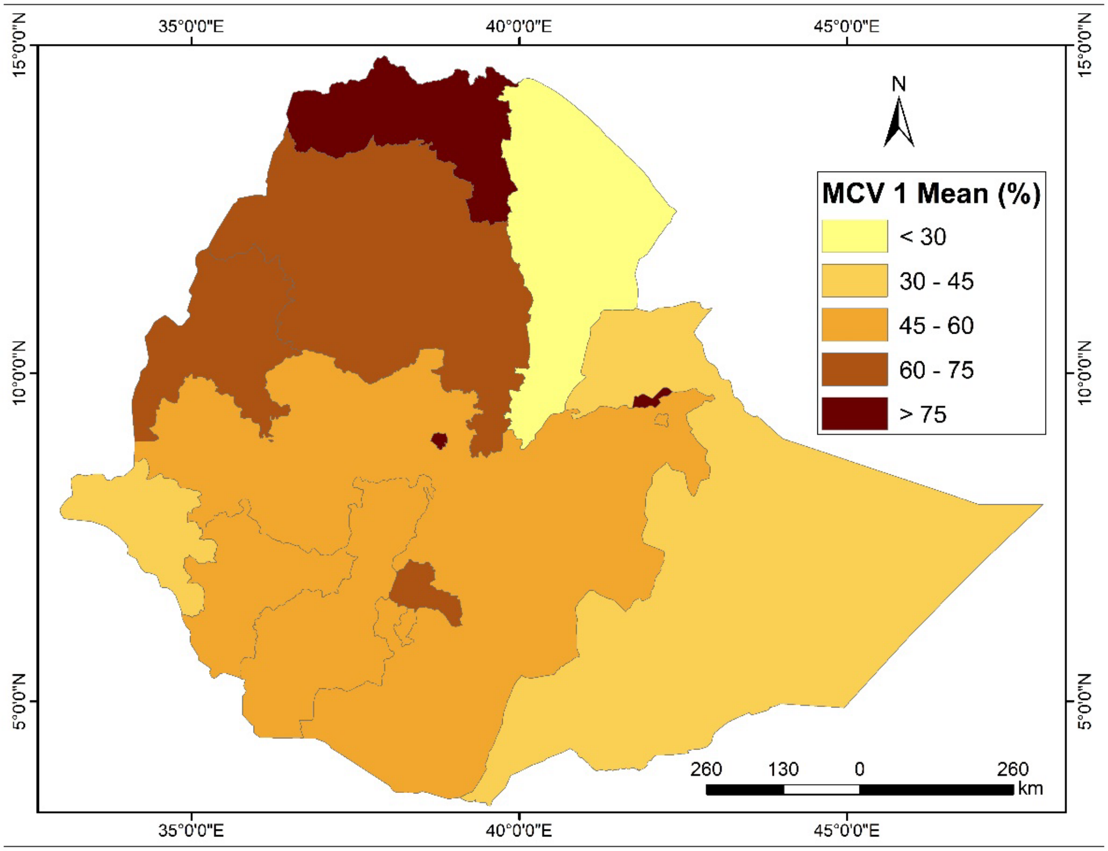

Figure A6.4. The third dose of diphtheria-pertussis-tetanus-Hib-HepB (Penta3) vaccine coverage for regions of Ethiopia from EDHS (2016 and 2019) surveys.

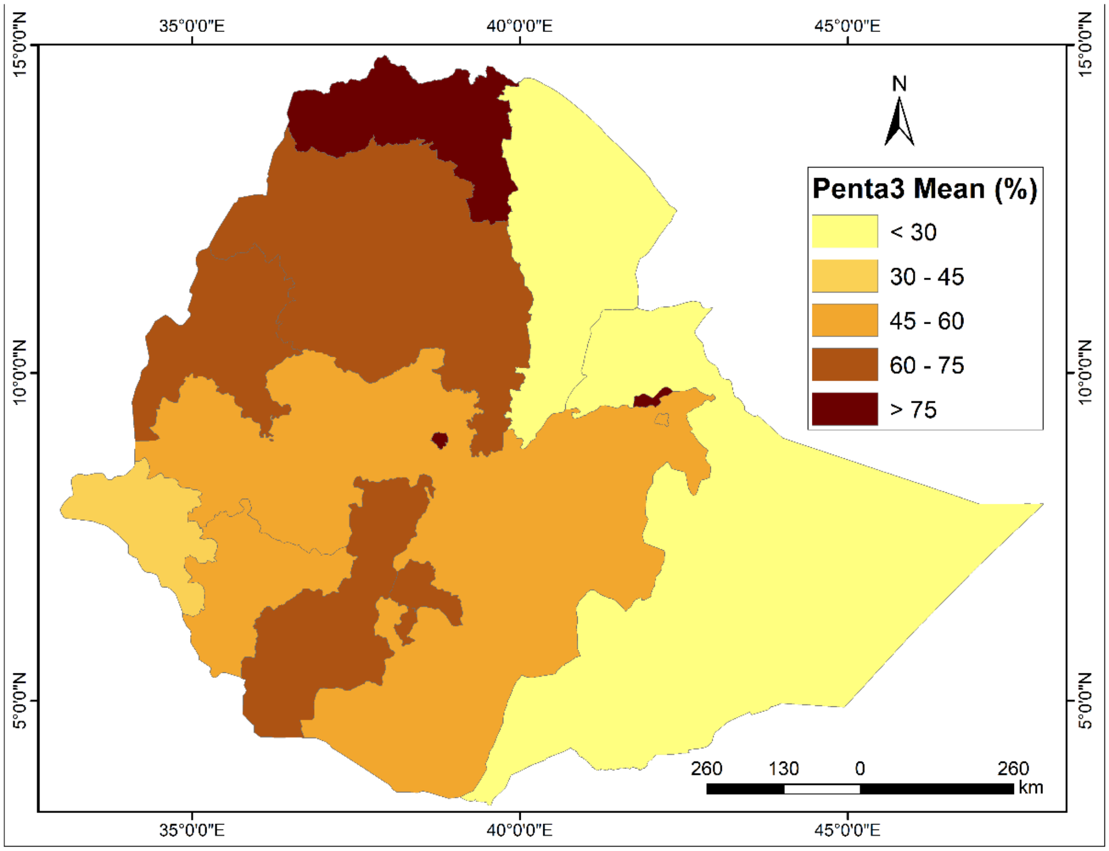

The above figures show MCV1 and Penta3 coverage for all the woredas included within EDHS 2016 and 2019 surveys. A 0% coverage indicates all the respondents of that specific woreda responded their children had not received the vaccine. However, since the data was disaggregated into one of the lowest administrative structures (i.e., woreda), the sample size was very small. The same issue arises for vaccine coverages that were 100%.

Addis Ababa, Harari, Sidama, and Dire Dawa regions did not have missing values across their woredas. However, the missing values across woredas of other regions varied widely. For woredas with missing values in Afar, the pattern of missingness for Penta3 ranged from 2% (missing value for one month) to 9%, whereas for MCV1 it ranged from 2 to 11%. For Amhara woredas, the missing value for Penta3 ranged from 2 to 25%, whereas for MCV1 it was 2 to 34%. For Benishangul-Gumuz, for both Penta3 and MCV1 it ranged from 3 to 26%. For Gambella, the missing values for Penta3 and MCV1 ranged from 2 to 33% and 2 to 36%, respectively. Similarly, missing values across Oromia woredas ranged from 2 to 5% for both Penta3 and MCV1. For Southern Nations Nationalities and Peoples, the missing values varied from 2 to 16% for Penta3, whereas for MCV1 it was from 2 to 18%. Somali had the most frequent monthly missing values across reporting woredas compared to any other region. This means there were multiple missing reports between months with data. Accordingly, Somali had the most woredas with missing values compared to any other region. For Penta3 this region had woredas with a missing value

ranging from 2 to 52%, and for MCV1 it was from 2 to 58%. Only 14 out of 98 woredas of this region were with no missing value. The Southwest Region had missing values ranging from 2 to 11% for Penta3 and from 2 to 21% for MCV1. Tigray had missing values from 7 to 52% for the Penta3 and from 7 to 56% for MCV1.

Table A6.1. Frequency of missing values across woredas for each region (showing the percentage and number of woredas within a given region with missing monthly report).

**Region/city administration**	**Percentage and number of woredas with missing values**	**Woreda with the largest number of missing values**
Addis Ababa	0% (n = 0)	-
Afar	26% (n = 10)	Bidu
Amhara	42% (n = 67)	Abergele
Benishangul Gumuz	50% (n = 11)	Yaso
Dire Dawa	0% (n = 0)	-
Gambella	36% (n = 5)	Akobo
Harari	0% (n = 0)	-
Oromia	1% (n = 339)	Nekemte City
Sidama	0% (n = 0)	-
Southern Nations Nationalities and Peoples	10% (n = 15)	Denba Gofa
Somali	86% (n = 84)	Gablalu
Southwest	6% (n = 3)	Surma
Tigray	4% (n = 2)	Adi Haki

Figure A6.5. Missing value patterns of Afar woredas. Left: Penta3, Right: MCV1.

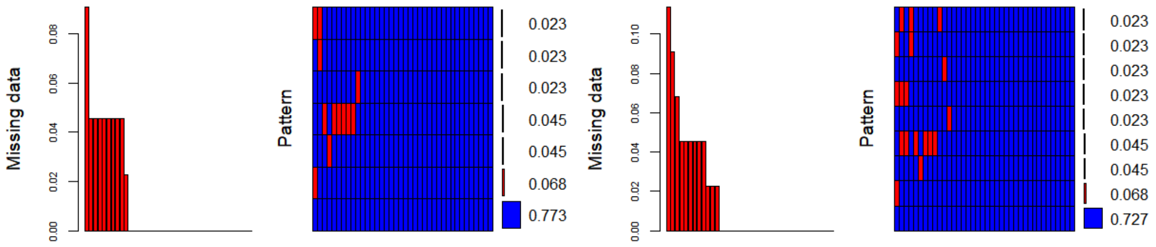

Note: The left panel illustrates the fractional representation of missing values across all woredas; the right panel delineates the monthly patterns of missing values for each woreda, with each row representing a distinct month. According to the data, 77% of woredas in Afar had complete records for Penta3 across all months, compared to 77% for MCV1.

Figure A6.6. Missing value patterns of Amhara woredas. Left: Penta3, Right: MCV1.

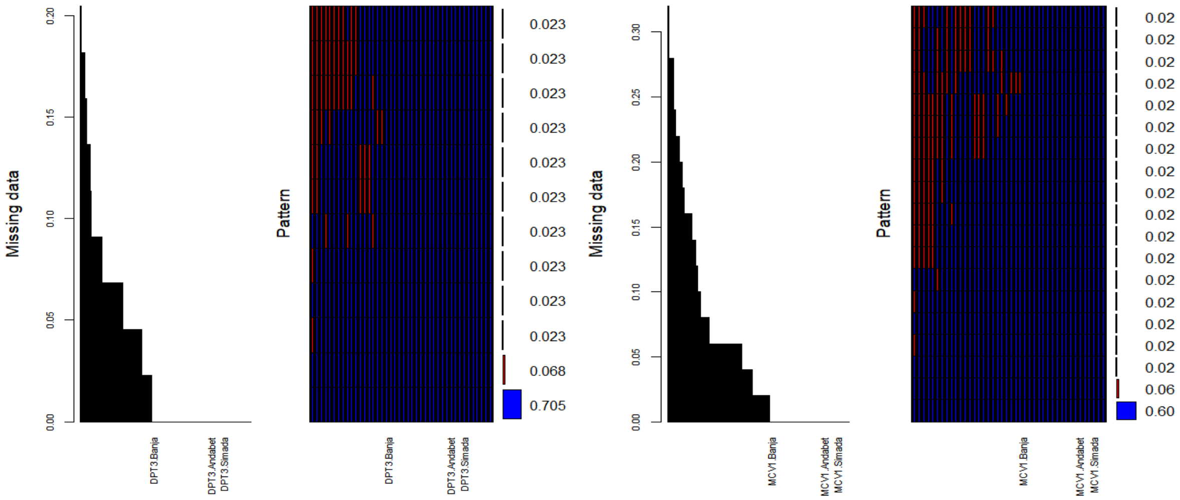

Note: The left panel illustrates the fractional representation of missing values across all woredas; the right panel delineates the monthly patterns of missing values for each woreda, with each row representing a distinct month. According to the data, 71% of woredas in Amhara had complete records for Penta3 across all months, compared to 60% for MCV1.

Figure A6.7. Missing value patterns of Benishangul-Gumuz woredas. Left: Penta3, Right: MCV1.

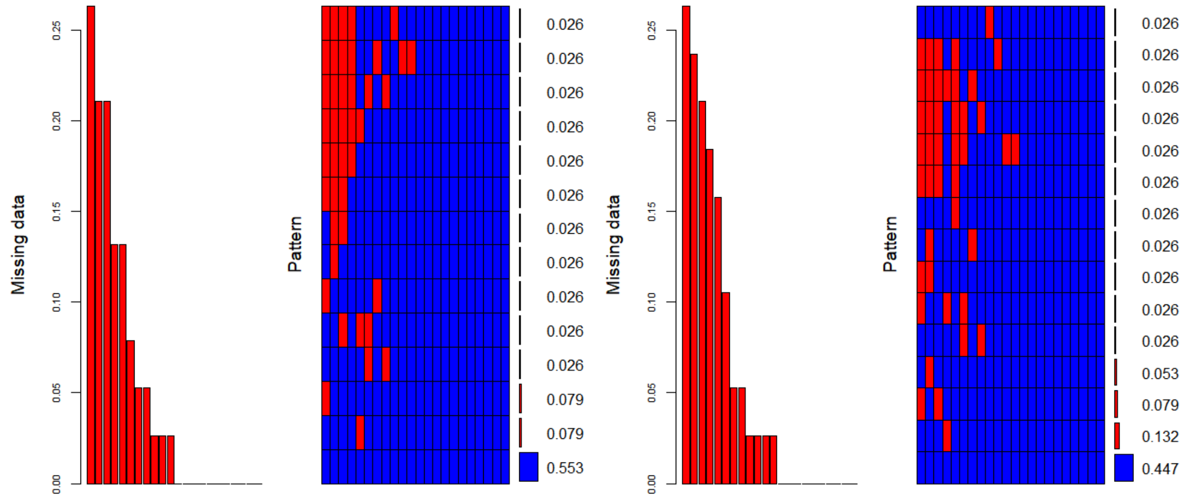

Note: The left panel illustrates the fractional representation of missing values across all woredas; the right panel delineates the monthly patterns of missing values for each woreda, with each row representing a distinct month. According to the data, 55% of woredas in Benishangul-Gumuz had complete records for Penta3 across all months, compared to 45% for MCV1.

Figure A6.8. Missing value patterns of Gambella woredas. Left: Penta3, Right: MCV1.

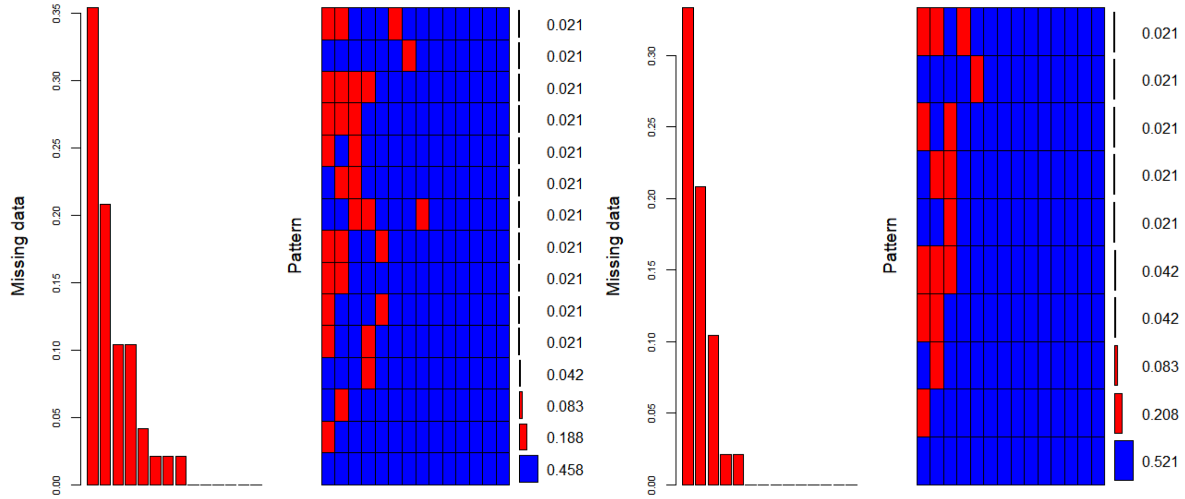

Note: The left panel illustrates the fractional representation of missing values across all woredas; the right panel delineates the monthly patterns of missing values for each woreda, with each row representing a distinct month. According to the data, 46% of woredas in Gambella had complete records for Penta3 across all months, compared to 52% for MCV1.

Figure A6.9. Missing value patterns of Oromia woredas. Left: Penta3, Right: MCV1.

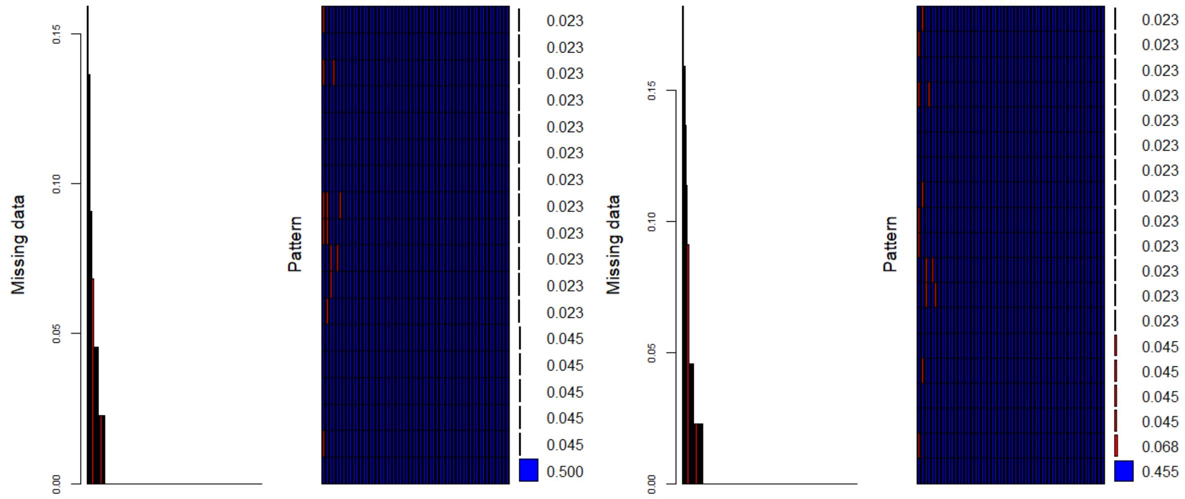

Note: The left panel illustrates the fractional representation of missing values across all woredas; the right panel delineates the monthly patterns of missing values for each woreda, with each row representing a distinct month. According to the data, 50% of woredas in Oromia had complete records for Penta3 across all months, compared to 46% for MCV1.

Figure A6.10. Missing value patterns of Somali woredas. Left: Penta3, Right: MCV1.

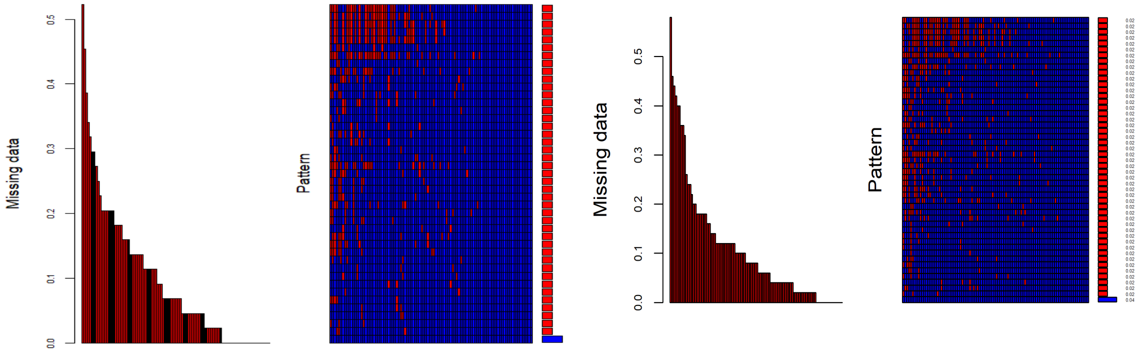

Note: The left panel illustrates the fractional representation of missing values across all woredas; the right panel delineates the monthly patterns of missing values for each woreda, with each row representing a distinct month. According to the data, 4% of woredas in Somali had complete records for Penta3 across all months, which is similar with MCV1.

Figure A6.11. Missing value patterns of South West Region woredas. Left: Penta3, Right: MCV1.

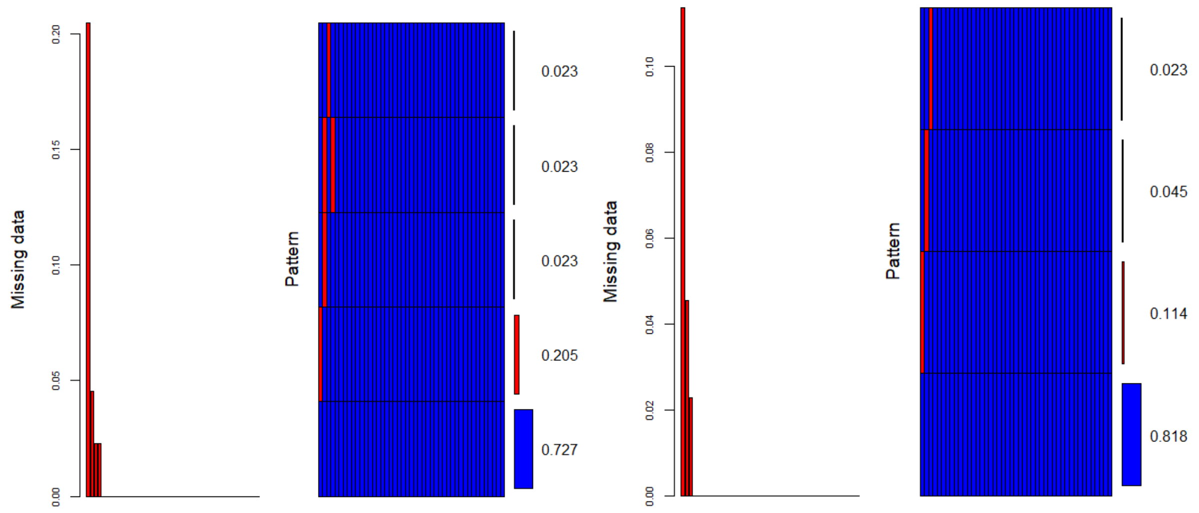

Note: The left panel illustrates the fractional representation of missing values across all woredas; the right panel delineates the monthly patterns of missing values for each woreda, with each row representing a distinct month. According to the data, 73% of woredas in South West Region had complete records for Penta3 across all months, compared to 82% for MCV1.

Figure A6.12. Missing value patterns of Tigray woredas. Left: Penta3, Right: MCV1.

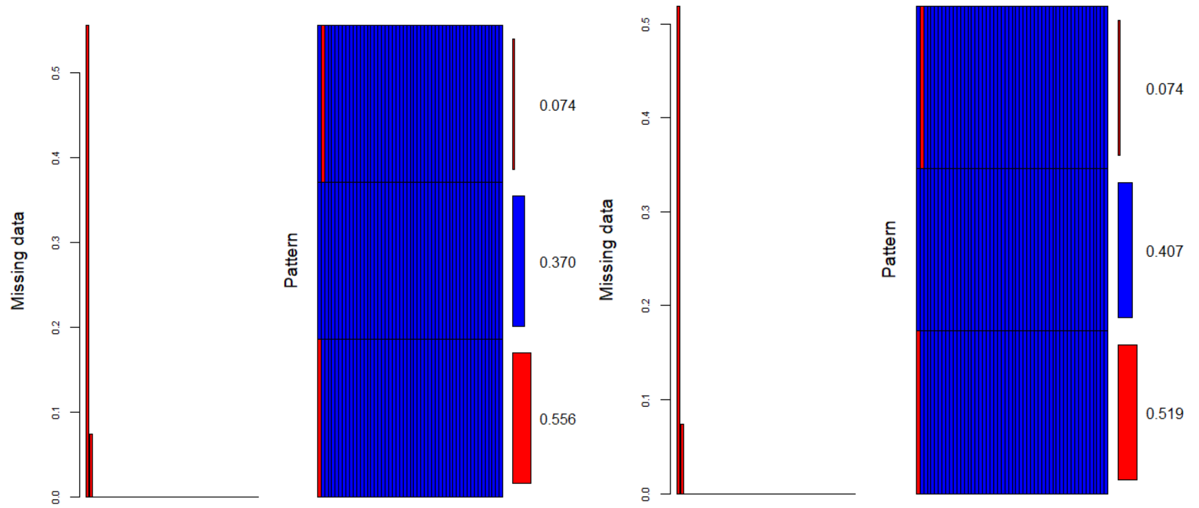

Note: The left panel illustrates the fractional representation of missing values across all woredas; the right panel delineates the monthly patterns of missing values for each woreda, with each row representing a distinct month. According to the data, 37% of woredas in Tigray had complete records for Penta3 across all months, compared to 41% for MCV1.

Figure A6.13. Missing value patterns of SNNPR woredas. Left: Penta3, Right: MCV1.

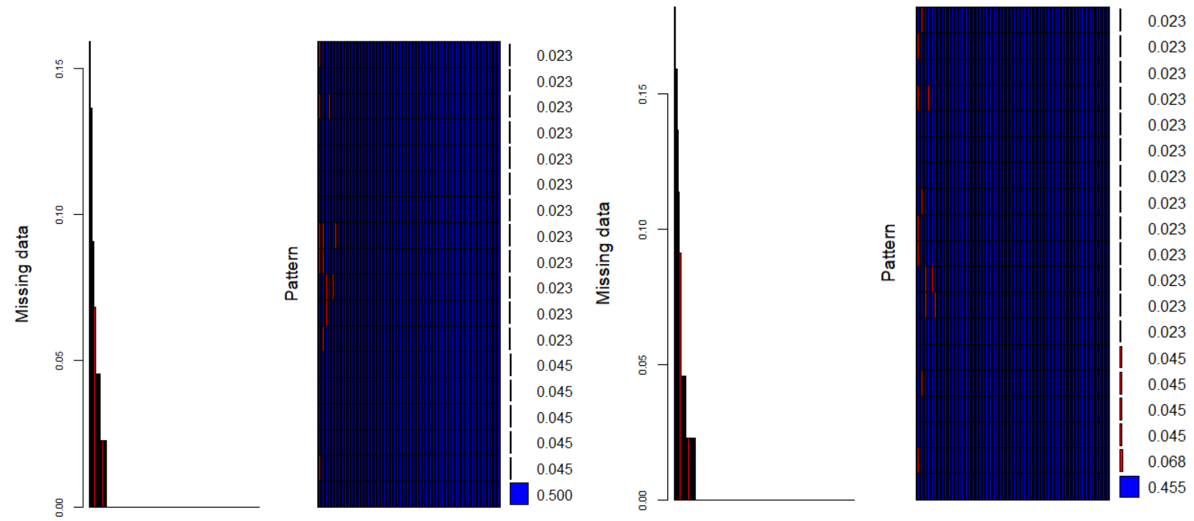

Note: The left panel illustrates the fractional representation of missing values across all woredas; the right panel delineates the monthly patterns of missing values for each woreda, with each row representing a distinct month. According to the data, 50% of woredas in SNNPR had complete records for Penta3 across all months, compared to 46% for MCV1.

Figure A6.14. Density plots of the imputed intermediate values: these figures compare the values generated during the imputation process with a given iteration (m=5, red graphs) with the blue graph (distribution of data before imputation). Selected woredas and Penta3 immunization are displayed.

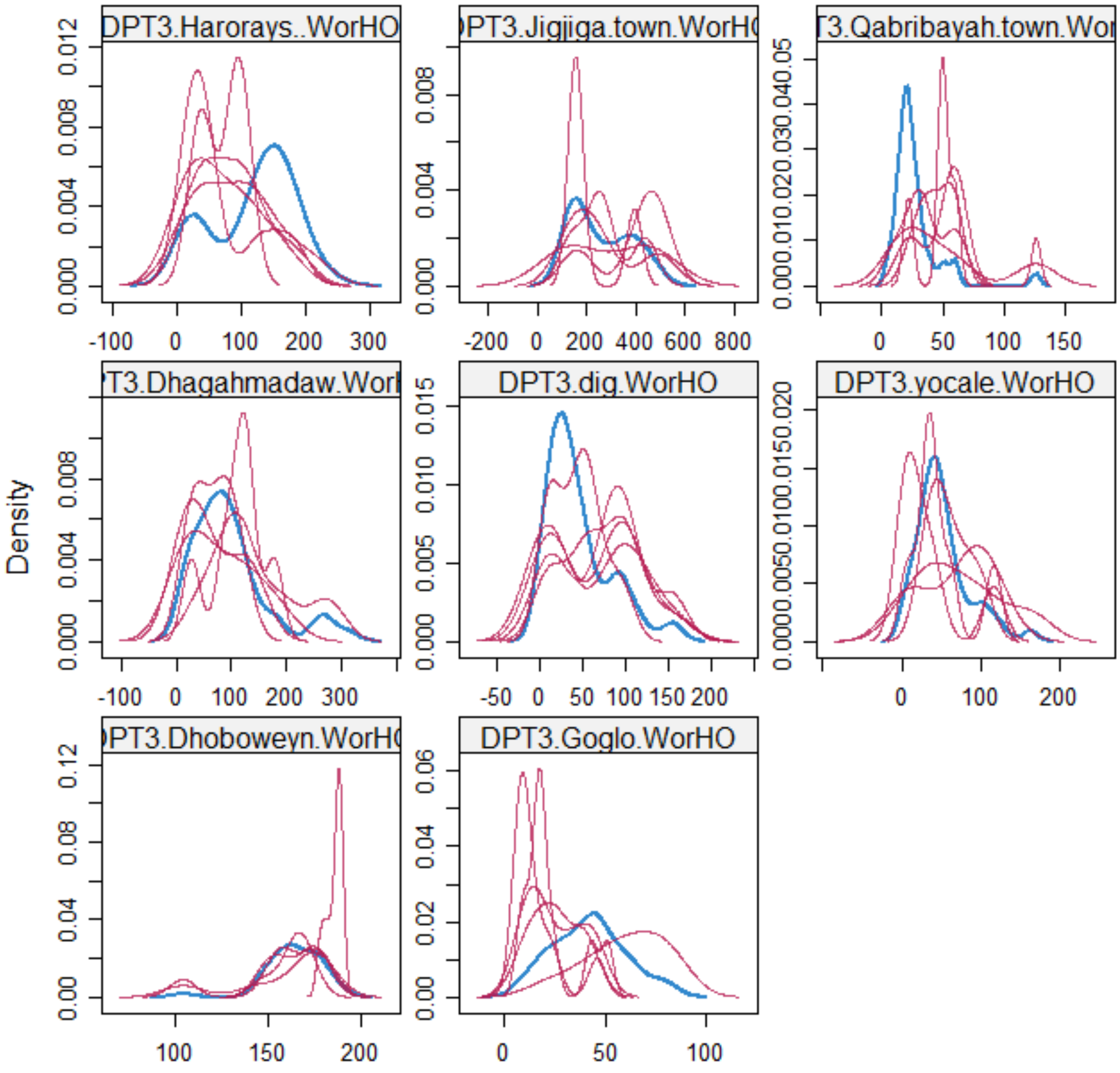

Table A6.2. Summary of missing values across woredas of each Ethiopian region.

Region/city administration	Percentage and number of woredas with outlier values	
	+3*SD	-3*SD
Addis Ababa	0% (n = 0)	0% (n = 0)
Afar	26% (n = 10)	21% (n = 8)
Amhara	42% (n = 67)	41% (n = 67)
Benishangul Gumuz	50% (n = 11)	32% (n = 7)
Dire Dawa	0% (n = 0)	0% (n = 0)
Gambella	36% (n = 5)	36% (n = 5)
Harari	0% (n = 0)	0% (n = 0)
Oromia	1% (n = 339)	0.9% (n = 328)
Sidama	0% (n = 0)	0% (n = 0)
SNNP	10% (n = 15)	7% (n = 10)
Somali	86% (n = 84)	77% (n = 75)
Southwest	6% (n = 3)	4% (n = 2)
Tigray	4% (n = 2)	2% (n = 1)

SD=Standard deviation; SNNP=Southern Nations, Nationalities, and Peoples.

Figure A6.15. Distributions of Penta3 data before and after adjustment for two selected woredas Angolala and Borena (Amhara).

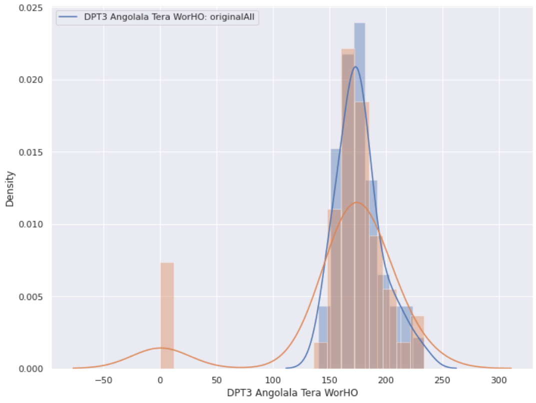

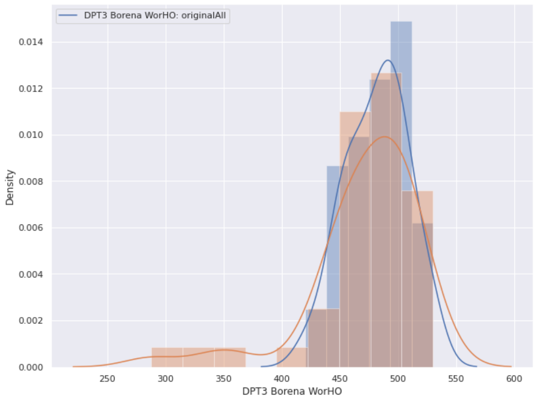

Note: The figures feature two distinct distributions for comparative analysis. The red distribution represents the original data prior any modification. The blue distribution represents estimates following imputation, outlier detection and subsequent treatment.

Figure A6.16. Scatter plot for adjusted vs. unadjusted coverage of Penta3 when using DHIS2 denominator.

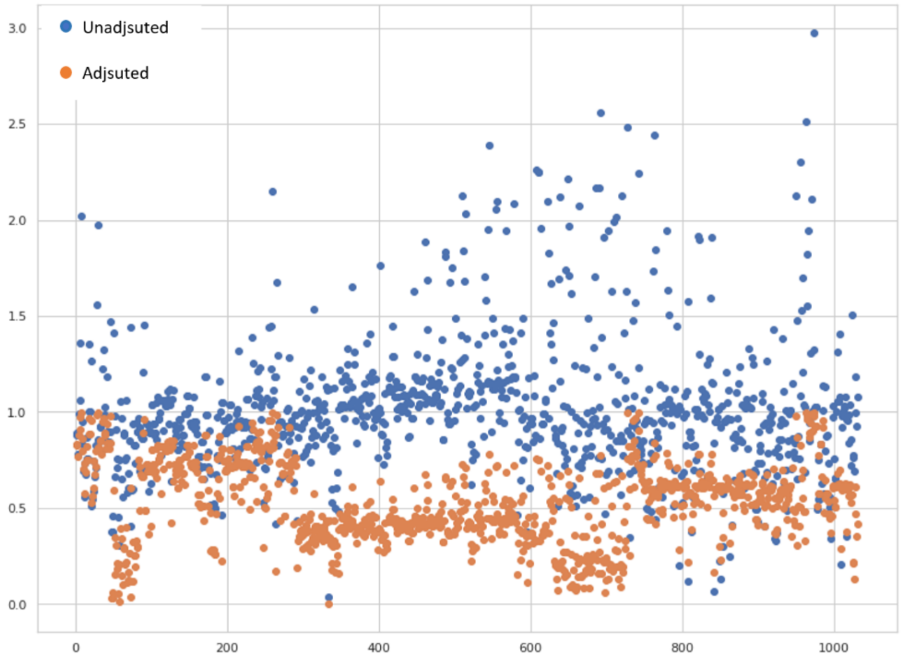

Figure A6.17. Scatter plot for adjusted vs. unadjusted coverage of Penta3 when using WorldPop denominator.

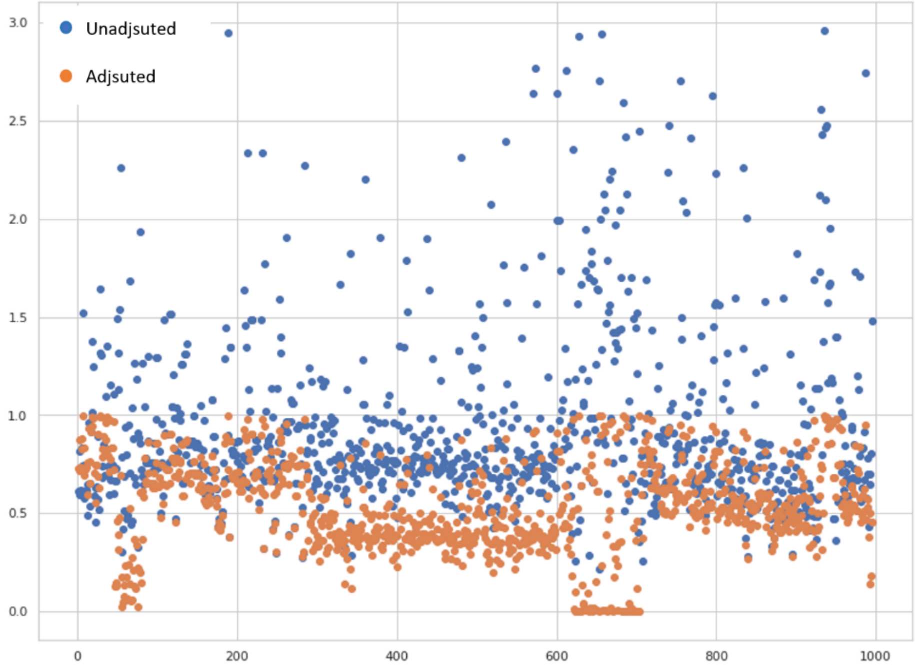

Supplement: S6 Text — (PDF) [file pgph.0003404.s006.pdf]
