# Supplementary material for: Estimating immunization coverage at the district level: A case study of measles and diphtheria-pertussis-tetanus-Hib-HepB vaccines in Ethiopia
Source: PLOS Glob Public Health. 2024 Jul 25;4(7):e0003404. doi: 10.1371/journal.pgph.0003404 (PMC11271922; doi:10.1371/journal.pgph.0003404)
Supplement: S7 Text — (PDF) [file pgph.0003404.s007.pdf]

S7 Text: Correcting administrative coverage with EDHS estimates

Our adjustment approach considers that districts could have arbitrarily high coverage rates and that ratios between administrative coverage and the actual values to be estimated are not necessarily constant. We create a model that maintains the observed ranking of districts based on their administrative coverage.

We fitted a continuous distribution for the administrative coverages. We observed the distribution in administrative coverage to be bell-shaped and right skewed. Therefore, we assumed a gamma distribution and used maximum-likelihood estimation to extract fitting parameters. Second, we needed a distribution for the adjusted (to be estimated) vaccine coverage for all districts, thus we matched the administrative coverage distribution to this hypothetical distribution, constrained to be bounded between 0 and 100%, and constrained to have an expectation set to the region-level coverage estimates from EDHS. This adjustment at the regional level was constrained to the ordering of districts within this distribution. The variance of the hypothetical distribution was based on the sample variance in district-level vaccine coverage.

We retrieved the percentiles of every observation in the original distribution. Then, we had to determine the parameters of our theoretical distribution. A beta distribution is usually characterized by its two shape parameters α and β. We estimated the shape parameters from the expectation and variance of the distribution as:

$$E[X] = \left(\frac{\alpha\beta}{\alpha+\beta}\right),$$

$$V[X] = \left(\frac{\alpha}{(\alpha+\beta)^2(1+\alpha+\beta)} - 1\right).$$

After solving for α and β we could obtain:

$$\alpha = \left(\frac{E[X](1-E[X])}{V[X]} - 1\right) E[X],$$

$$\beta = \left(\frac{E[X](1-E[X])}{V[X]} - 1\right) (1 - E[X]).$$

We needed α and β to characterize the Beta distribution. As mentioned above, we took $E[X]$ to be the EDHS regional coverage estimates and $V[X]$ to be the variance in woreda vaccine coverage across regions.
