# Supplementary material for: Estimating immunization coverage at the district level: A case study of measles and diphtheria-pertussis-tetanus-Hib-HepB vaccines in Ethiopia
Source: PLOS Glob Public Health. 2024 Jul 25;4(7):e0003404. doi: 10.1371/journal.pgph.0003404 (PMC11271922; doi:10.1371/journal.pgph.0003404)
Supplement: S8 Text — (PDF) [file pgph.0003404.s008.pdf]

S8 Text: Immunization disparities across woredas in Ethiopia

To assess disparities in the number of vaccines administered, we generated a modified Lorenz curve to assess disparities in Penta3 and MCV1 coverage.

A conventional method of generating the Lorenz curve and Gini index can be modified to account for the statistical distribution of the data. The Lorenz curve and Gini index were generated separately for 13 Ethiopian regions and at the national level, by using the cumulative target population percentage of the woredas (x-axis, 0-100%) and the cumulative percentage of the actual number of vaccines administered (y-axis, 0-100%).

Figure A8.1. Simple sketch of a hypothetical Lorenz curve.

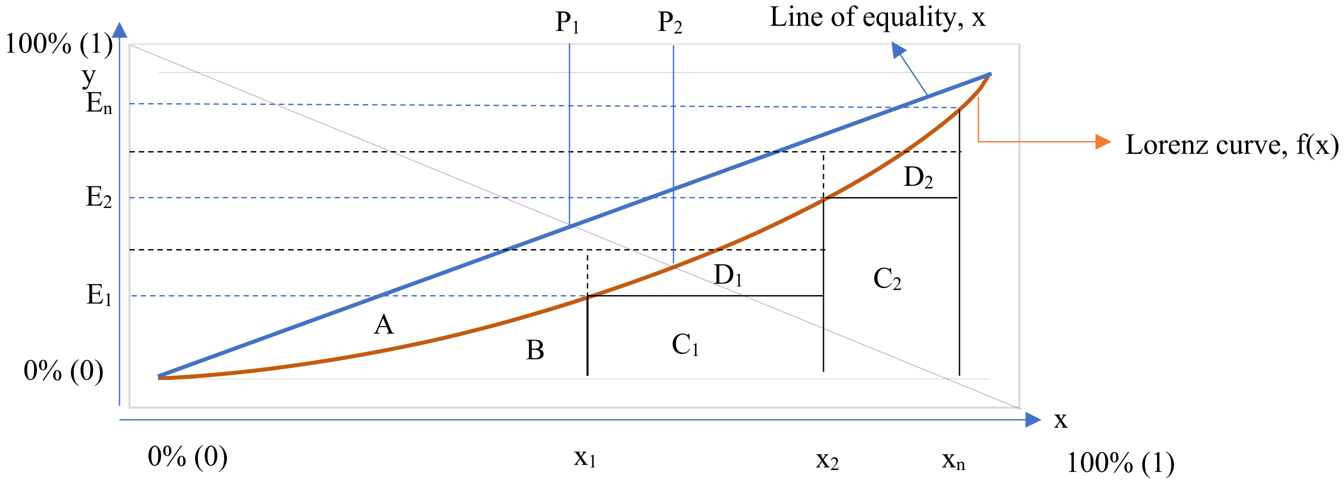

Option 1 - Geometric estimation

As shown (Figure A8.1), smaller rectangular areas of D and C series can be used to estimate the area under the Lorenz curve i.e. B, and then the area between the line of equality and the Lorenz curve i.e. A. The height of these smaller rectangles can be estimated from values of $\{E_1, E_2 ..... E_n\}$, which is the cumulative percentage of vaccines administered. Their width can be estimated from values of $\{x_1, x_2 ..... x_n\}$. Assuming a symmetric Lorenz curve, each width can be estimated as the inverse of the total number of observations. This means: if we have a total of n observations, the widths can be estimated as 1/n. The Gini index is defined as the ratio of area A and area A + B (4). The sum of areas A and B equals ½ * (width = 1 * height = 1). Accordingly, the Gini index is twice area A or 1 – 2*B. One apparent challenge here is that, if we use rectangular series of D to estimate A, it will be underestimated. On the other hand, if we use rectangular series of area C, it will be overestimated. Therefore, we use the average of the two rectangular areas to estimate area A. From the above figure, for instance area $D_1 = (E_2 – E1)/(2*x)$, whereas $C_1 = E_1*1/x$. The average of the two areas will give us $(E_2 + E1)/(2*x)$. This value represents the area under the Lorenz curve partially for D_1 and fully for C_1. Using a similar method for other rectangular areas of C's and D's, and summing up their values, give us a total area of B. Then the area of A is the area

of B minus 1/2 or 50%, depending on the x-axis and y-axis scale. Finally, the Gini index can be calculated by either of the above formulas. The above procedures can be summarized with the following formula:

$$G\ (Gini\ index) = \frac{1}{x} * (x + 1 - 2 * \left(\frac{\sum_{i=1}^{x}(x + 1 - i) * Ei}{\sum_{i=1}^{x} Ei}\right)). \quad (1)$$

This method is applicable for small observations (x) and when it is difficult to explain the Lorenz curve with a continuous function.

Option 2 - Estimate the Lorenz curve for continuous distributions

If the Lorenz curve can be estimated or fitted to a continuous function f(x), area B can be calculated with the following integral formula:

$$B(x) = \int_0^1 f(x)dx\ . \quad (2)$$

Accordingly, the Gini index or area of A can be calculated as:

$$G = 1 - 2 * \int_0^1 f(x)dx. \quad (3).$$

If we have a large population or observations and the E values (here vaccines administered) can be explained as a continuous distribution *L(x)*, with probability density function pdf(x), then the Gini index can be calculated using:

$$G = 1 - 2 * \int_0^1 L(x)dx\ . \quad (4)$$

The Lorenz curve *L(x)* for a given distribution can be estimated as:

$$L(x) = \frac{1}{\lambda}\int_0^x \left(x * pdf(x)\right)\ . \quad (5)$$

The first step is to find a distribution best fitting the data (number of vaccines administered across woredas). A Python library called Fitter is used to find a best fitting distribution from Gamma, Weibull, t-distribution, Exponential, Uniform, Pareto, Log-gamma, Log-normal, Beta, Burr, and Normal distributions [1]. A distribution with the least Sum Squared Error (SSE) was selected as a distribution function for *L(x)*. The Beta distribution had the lowest SSE. The pdf(x) and λ of Beta distribution for 0<x<1 and shape parameters α, β > 0 are given by:

$$pdf(x) = \frac{1}{B(\alpha, \beta)} x^{\alpha - 1} * (1 - x)^{\beta - 1}\ , \quad (6)$$

where $B(\alpha, \beta)$ is a Beta function and estimated by using the Gamma function (4):

$$B(\alpha, \beta) = \frac{\Gamma(\alpha) * \Gamma(\beta)}{\Gamma(\alpha + \beta)}\ . \quad (7)$$

The mean (λ) is given as $\frac{\alpha}{(\alpha+\beta)}$. Substituting into equation (5) and solving the integral between [0;1], the Gini index becomes:

$$G = \frac{2}{\alpha} * \frac{B(\alpha + \beta, \alpha + \beta)}{B(\alpha, \alpha) * B(\beta, \beta)} . \quad (8)$$

The Gini index ranges from 0 to 1, with 0 indicating perfect equality and 1 perfect inequality [2].

The above two options were used depending on how well data for a given region could fit the continuous distribution functions. For regions with a small number of woredas, the SSE was found to be higher compared to regions with a large number of woredas. For Addis Ababa, Harari, and Dire Dawa the first option was used. For other regions and at the national level the second option was used.

Figure A8.2. Fitting the data (number of Penta3 vaccines) against common distributions.

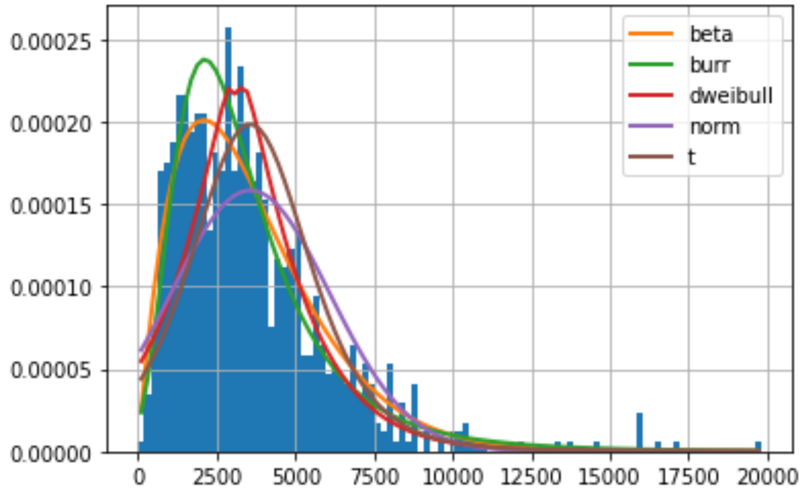

As Figure A8.2 shows, the Beta distribution was found to be a good fit with the smallest SSE for both MCV1 and Penta3. Using the geometric option we found the Gini index to be 0.38 at the national level for Penta3. Finally, for the Beta distribution, the Gini index was calculated to be 0.36. Both methods yielded close results.

Table A8.1. Gini coefficient of regions. *L(x)* is assumed to follow a beta distribution.

Region	Gini index - Penta3	Gini index - MCV1
Addis Ababa	0.233	0.229
Afar	0.231	0.233
Amhara	0.274	0.278
Harari	0.158	0.130
Gambella	0.283	0.281
Benishangul Gumuz	0.275	0.304
Sidama	0.297	0.301
Dire Dawa	0.283	0.265
Tigray	0.280	0.282
SNNP	0.266	0.266
Oromia	0.330	0.327
SWR	0.317	0.317
Somali	0.356	0.368
National	0.363	0.366

References

1. Cokelaer T, Kravchenko A, Varma ALB, Stringari CE, Brueffer C, Broda E, Pruesse E, Singaravelan K, & Padgham M. (2022). *cokelaer/fitter: v1.5.1*. https://doi.org/10.5281/ZENODO.7080297
2. Tao Y, Henry K, Zou Q, Zhong X. Methods for measuring horizontal equity in health resource allocation: a comparative study. Health Econ Rev. 2014;4(1):1-10.
